# Exosomal circLPAR1 functions in colorectal cancer diagnosis and tumorigenesis through suppressing *BRD4* via METTL3–eIF3h interaction

**DOI:** 10.1186/s12943-021-01471-y

**Published:** 2022-02-14

**Authors:** Rui Zheng, Ke Zhang, Shanyue Tan, Fang Gao, Yajie Zhang, Wenxia Xu, Huabin Wang, Dongying Gu, Lingjun Zhu, Shuwei Li, Haiyan Chu, Zhengdong Zhang, Lingxiang Liu, Mulong Du, Meilin Wang

**Affiliations:** 1 Department of Environmental Genomics, Jiangsu Key Laboratory of Cancer Biomarkers, Prevention and Treatment, Collaborative Innovation Center for Cancer Personalized Medicine, School of Public Health, Nanjing Medical University, 101 Longmian Avenue, Jiangning District, Nanjing, 211166 China; 2 Department of Genetic Toxicology, The Key Laboratory of Modern Toxicology of Ministry of Education, Center for Global Health, School of Public Health, Nanjing Medical University, 101 Longmian Avenue, Jiangning District, Nanjing, 211166 China; 3 Department of Oncology, The First Affiliated Hospital of Nanjing Medical University, 300 Guangzhou Road, Nanjing, 210029 China; 4 Key Laboratory of Environmental Medicine Engineering, Ministry of Education of China, School of Public Health, Southeast University, Nanjing, China; 5 Department of Central Laboratory, The Affiliated Nanjing Hospital of Nanjing University of Chinese Medicine, Nanjing, China; 6 Department of Clinical Biobank, The Affiliated Nanjing Hospital of Nanjing University of Chinese Medicine, Nanjing, China; 7 Central Laboratory, Affiliated Jinhua Hospital, Zhejiang University School of Medicine, Jinhua, China; 8 Department of Oncology, Nanjing First Hospital, Nanjing Medical University, Nanjing, China; 9 Department of Biostatistics, Center for Global Health, School of Public Health, Nanjing Medical University, 101 Longmian Avenue, Jiangning District, Nanjing, 211166 China; 10 The Affiliated Suzhou Hospital of Nanjing Medical University, Suzhou Municipal Hospital, Gusu School, Nanjing Medical University, Suzhou, China; 11 Jiangsu Cancer Hospital, Jiangsu Institute of Cancer Research, The Affiliated Cancer Hospital of Nanjing Medical University, Nanjing, China

**Keywords:** Colorectal cancer, Exosomal circLPAR1, Biomarker, METTL3–eIF3h interaction, *BRD4*

## Abstract

**Background:**

Exosomes have emerged as vital biomarkers of multiple cancers and contain abundant circular RNAs (circRNAs). However, the potential for exosomal circRNAs to be used in diagnostics and their molecular mechanism of action in colorectal cancer (CRC) remain unclear.

**Methods:**

CRC-specific exosomal circRNAs were identified by RNA sequencing, exoRBase database and a tissue microarray. The diagnostic performance of plasma exosomal circRNAs was evaluated among cancer-free controls, precancer individuals, CRC patients, and patients with other types of cancer. The corresponding biological functions were mainly assessed using circRNA pull-down, proteomic analysis, and RNA immunoprecipitation assay underlying cellular and mouse models.

**Results:**

CircLPAR1 was encapsulated in exosomes with high stability and detectability, and its expression in plasma exosomes was remarkably decreased during CRC development but recovered after surgery. Exosomal circLPAR1 showed cancer specificity in CRC diagnosis and increased the diagnostic performance to an area under the receiver operating characteristic curve of 0.875, as determined by analysing its performance in combination with common clinical biomarkers CEA and CA19–9. Additionally, circLPAR1 was downregulated in CRC tissues and was associated with overall survival. Mechanistically, exosomal circLPAR1 was internalized by CRC cells, and it suppressed tumor growth, likely because exosomal circLPAR1 directly bound with eIF3h specifically suppressed the METTL3-eIF3h interaction, decreasing the translation of oncogene *BRD4*.

**Conclusions:**

This comprehensive study highlights plasma exosomal circLPAR1 as a promising predictor in CRC diagnosis and describes its biological regulation of colorectal tumorigenesis. This study provides a new perspective on early diagnosis in the clinic and pathogenesis in disease development.

**Supplementary Information:**

The online version contains supplementary material available at 10.1186/s12943-021-01471-y.

## Background

Colorectal cancer has emerged as one of the most prevalent malignancies of the digestive system [1], and in 2020, more than 1.9 million new colorectal cancer cases and 935,000 deaths were estimated to have occurred worldwide [2]. In China, the morbidity and mortality of colorectal cancer have remained high in recent years [3]. Although some approaches have been applied in the clinic, including colonoscopy, sigmoidoscopy and faecal-based testing, the early detection and diagnosis of colorectal cancer remain challenging [4, 5]. Thus, there is an urgent need to identify novel biomarkers of colorectal cancer.

Exosomes, which are regarded as a valuable source of promising biomarkers, have a size range of 40-120 nm and exist stably in bodily fluids [6, 7]. Abundant evidence has shown that exosomes act as messengers in cell-to-cell communication by delivering specific cargos derived from parent cells to recipient cells involved in disease development and progression [8]. Recently, circular RNAs (circRNAs) have been shown to be definitively enriched in exosomes and are easy to measurement in the circulation, and with covalently closed loop structures without 5′ to 3′ polarity or a polyadenylated tail, they are highly stable compared with their parental linear RNAs [9–11]. Moreover, circRNAs can be specifically and differentially expressed under various pathologic conditions across tissues and fluids, including colorectal cancer [12, 13], thus serving as potential biomarkers of disease progression for use in diagnostics [14, 15]. Accordingly, exosomal circRNAs are novel in the frontier of cancer biomarkers with promise for use in both clinical applications and studies of disease aetiology [11]. Currently, few studies have reported on the diagnostic role of exosomal circRNA in colorectal cancer [16, 17]. For example, Shang et al. found that exosomal circPACRGL plays an oncogenic role in colorectal cancer and could be a potential marker for colorectal cancer [18]. However, the validation sample size of these studies is limited, and the role of exosomal circRNAs as a new biomarker for colorectal cancer has not been systematically studied.

In this study, we initially performed high-throughput RNA sequencing (RNA-Seq) of tissues, and the results were validated through tissue microarray, cell line and exosome analyses. Subsequently, we evaluated the diagnostic capabilities of target exosomal circRNAs in plasma obtained from cancer-free controls, precancer individuals, colorectal cancer patients and patients with other types of cancer. Furthermore, in vitro and in vivo experiments with cell and mouse models were conducted to comprehensively clarify the biological function of a target exosomal circRNA involved in colorectal tumorigenesis.

## Materials and methods

### Study populations

Patients with colorectal cancer (*n* = 112) or polyps (*n* = 28) were randomly selected from the Nanjing ColoRectal Cancer (NJCRC) cohort, which is a long-term follow-up clinical cohort [19, 20]. Briefly, the NJCRC study followed inclusion criteria indicated that participants must have been recently diagnosed by clinical and histopathology and not treated for colorectal cancer patients during surgical resection. Cancer-free control individuals (*n* = 60) were recruited from the same geographical region during the same period in which other study participants were recruited. Moreover, patients with other cancers were consecutively recruited from the Collaborative Innovation Center for Cancer Personalized Medicine of Nanjing Medical University, including patients with gastric carcinoma (GC, *n* = 74), breast invasive carcinoma (BRCA, *n* = 18), bladder urothelial carcinoma (BLCA, *n* = 24), cervical squamous cell carcinoma and endocervical adenocarcinoma (CESC, *n* = 32), kidney renal clear cell carcinoma (KIRC, *n* = 19), and lung adenocarcinoma (LUAD, *n* = 42). Corresponding tumor/normal tissues and peripheral blood samples were collected, and plasma was obtained by centrifuging peripheral blood at 3000 rpm at 4 °C for 10 min. We also collected plasma samples from 25 colorectal cancer patients before and after surgical treatment. Written informed consent was obtained from all subjects recruited for this study, and the study was authorized by the Institutional Review Board of Nanjing Medical University (approval No. 2017-515).

### Transcriptomics and proteomic analysis

A total of 52 pairs of colorectal tumors and normal adjacent tissues (NATs) were subjected to RNA-Seq to identify differentially expressed circRNAs. The circRNAs profiled were sequenced and generated with an Illumina HiSeq 2500 platform for 150-bp sequencing in collaboration with Genesky Biotechnologies, Inc. (Shanghai, China). The Find_circ [21] and CIRI2 [22] algorithms were used to detect and identify circRNAs. The raw counts were normalized on the basis of transcripts per kilobase million (TPM), and the differential expression profiles of the circRNAs were obtained using the R package “edgeR”. The TPM normalization method was used to normalize the RNA-Seq data because this method respects the average invariance and prevents the biological signal compared with other normalization methods [23–25]. In addition, a tandem mass tag (TMT)-based quantitative proteomic analysis (Novogene Co., Ltd., China) was performed to identify and quantitate proteins based on 25 pairs of colorectal tumors and NATs (the samples were from RNA-Seq samples).

### Fluorescence in situ hybridization (FISH) and immunofluorescence

FISH with 5-carboxyfluorescein (5-FAM)-labelled probe sequences specific for circLPAR1 (GenePharma, China) was performed to detect the expression and visualize the localization of circLPAR1 in a tissue microarray with 79 paired colorectal tumor tissues and NATs. The probe was dropped onto slides, and hybridization was performed overnight at 60 °C in a moist chamber. After hybridization, the slides were washed twice once with 2 × saline-sodium citrate (SSC) for 5 min, once in 0.5 × SSC for 15 min, and twice in 0.2 × SSC for 15 min. The slides were then subsequently fluorescein isothiocyanate (FITC)-streptavidin-biotin complex (SABC) at 37 °C for 45 min. The immunofluorescence accumulation optical density (IOD) of circLPAR1 was evaluated by Image-Pro Plus 7.0 software (Media Cybernetics, Inc., USA) [26]. In addition, RNA-FISH was carried out by using a FISH Kit (RiboBio Inc., China) to assess the location of circLPAR1 in colorectal cancer cells. To observe the colocalization of circLPAR1 and eukaryotic translation initiation factor 3 subunit h (eIF3h), DLD1 cells were transiently transfected with Cyanine3 (Cy3)-labelled circLPAR1 (GenePharma, China) and then incubated with anti-eIF3h antibody.

### Exosome isolation, characterization, and internalization

Exosomes were isolated from the culture medium of FHC, HCT116 and DLD1 cells by using an ExoQuick TC kit (SBI, USA), and exosomes from the plasma of subjects were purified by using an ExoQuick Plasma Prep with Thrombin kit (SBI, USA). The size distribution of the exosomes was characterized by nanoflow cytometry using a U30 Flow NanoAnalyzer (NanoFCM, Inc., China) with technical assistance provided by KeyGEN Biotech Co. Ltd. (Jiangsu Province, China). The shape and size of the exosomes were observed by transmission electron microscopy (TEM) (Tecnai G2, FEI, USA). Moreover, the characterization of the exosomes was confirmed by the presence of exosomal protein markers TSG101 (# ab125011, Abcam, USA) and Alix (# 92880, CST, USA). The green fluorescent dye PKH67 (Umibio, China) was utilized to label exosomes isolated from the culture medium of cells. After dye was incubated with recipient cells for 3 h, fluorescence microscopy (Zeiss, Germany) was performed to visualize PKH67-labelled exosomes in recipient cells. The detailed procedures were described in our previous study [27].

### Stability determination of plasma exosomal circLPAR1

To determine the stability of circLPAR1, colorectal cancer cells were treated with 6 U/6 μg RNase R (Epicentre Biotechnologies, USA) at 37 °C for 10 min. Moreover, 2 μg/ml actinomycin D (AbMole, China) was added to colorectal cancer cells and incubated for 1 h, 3 h, 6 h, and 24 h to inhibit new RNA synthesis. To determine the stability of plasma exosomal circLPAR1, plasma samples from subjects (colorectal cancer patients, precancer individuals and cancer-free control individuals) were subjected to extreme conditions, including incubation at room temperature for 0 h, 4 h, 8 h, and 24 h and repeated cycles (0, 2, 4, and 8 cycles) of freezing and thawing between − 80 °C and room temperature.

### CircLPAR1 pull-down assay and mass spectrometry analysis

The RNA-binding proteins (RBPs) associated with circLPAR1 were determined by using a circRNA pull-down assay with MS2-capturing protein (MS2-CP) [28, 29]. Briefly, two overexpression vectors, one carrying circLPAR1-MS2 and the other carrying MS2-CP-Flag, were constructed and labelled with green fluorescent protein (GFP) and red fluorescent protein (m-Cherry), respectively (Geneseed, China). These two vectors were co-transfected into HCT116 cells to induce MS2-CP expression. After specific binding between MS2-labelled circLPAR1 and MS2-CP, the MS2-CP-MS2-circLPAR1 complex was pulled down by an anti-Flag antibody. Lysate derived from HCT116 cells without the MS2 flagging system was used as the control. Subsequently, the captured products were identified by real-time quantitative polymerase chain reaction (RT-qPCR) with a fluorescent reporter or Western blotting. Then, the circLPAR1 pull-down complex and its control were analysed by mass spectrometry (Q Exactive, Thermo Scientific, USA).

### RNA immunoprecipitation (RIP) assay

The RIP assay was performed with a Magna RIP kit (Millipore Magna, USA) to determine the interaction between exosomal circLPAR1 and eIF3h. In brief, lysates of HCT116 and DLD1 cells with stable overexpression of circLPAR1 or exosomal circLPAR1 were incubated with RIP buffer containing anti-eIF3h antibody (# 3413, CST, USA)- or control IgG (# ab172730, Abcam, USA)-conjugated magnetic beads. The immunoprecipitated RNA was then analysed by RT-qPCR.

### Effects of exosomal circLPAR1 on mouse models of colorectal cancer

A total of 24 female NCG mice (4–5 weeks old) were randomly divided into the following four groups: NC; circLPAR1; circLPAR1-Exos; and NC-Exos. On days 1, 3 and 5, 1 × 10^7^ circLPAR1 or NC stably expressed DLD1 cell lines were resuspended in 0.1 ml of phosphate-buffered saline (PBS) and injected into the right flank of the mice. PBS was injected into mouse tumors 15, 17 and 19 days after mouse tumor induction, and these tumors were used as the NC and circLPAR1 groups, respectively. To assess the effect of exosomal circLPAR1 on colorectal cancer tumorigenesis, an equal DLD1 cell concentration was injected into the right flank of normal mice on days 1, 3 and 5. Exosomes isolated from DLD1 cells stably transfected with circLPAR1/NC vector were injected into mouse tumors 15, 17 and 19 days after tumor induction, and these tumors were used as the circLPAR1-Exos and NC-Exos groups, respectively. The width and length of the tumors in each group were measured once every 2 days, and the tumor volume was calculated using the following formula: tumor volume (cm^3^) = (*L* × *W*^2^)/2, where *L* is length and *W* is width. All animal studies were approved by the Institutional Animal Care and Use Committee of Nanjing Medical University (IACUC-2012051).

### In silico analyses

The circBase database (http://circrna.org/) was utilized to annotate circRNAs [30] and the exoRBase 1.0 database in 2018 (http://www.exoRBase.org) was used to annotate exosomal circRNAs [31]. The catRAPID signature/express module (http://service.tartaglialab.com/page/catrapid_group) was performed to estimate the binding capacity of RBPs with circLPAR1 [32, 33].

### Statistical analysis

Quantitative data are presented as the means ± standard deviations (SDs) and the group comparison was evaluated by Student’s *t*-test (two-tailed). Unconditional univariate Cox regression analysis was utilized to obtain hazard ratios (HRs) and Kaplan-Meier analysis was used to evaluate the effect of circLPAR1 on the overall survival of colorectal cancer patients. The receiver operating characteristic (ROC) curve analysis was used to calculate the area under the curve (AUC) value to assess the capability for discriminating colorectal cancer patients from cancer-free controls. The correlation between circLPAR1 and RBPs in human tissues was determined with Spearman’s correlation analysis. A two-sided *P* < 0.05 was considered statistically significant. Statistical analyses were performed with R software (version 3.6.0). Additional experimental methods are described in the Supplementary Materials and Methods.

## Results

### Downregulated circLPAR1 is encapsulated in exosomes in colorectal cancer

**Fig. 1:**
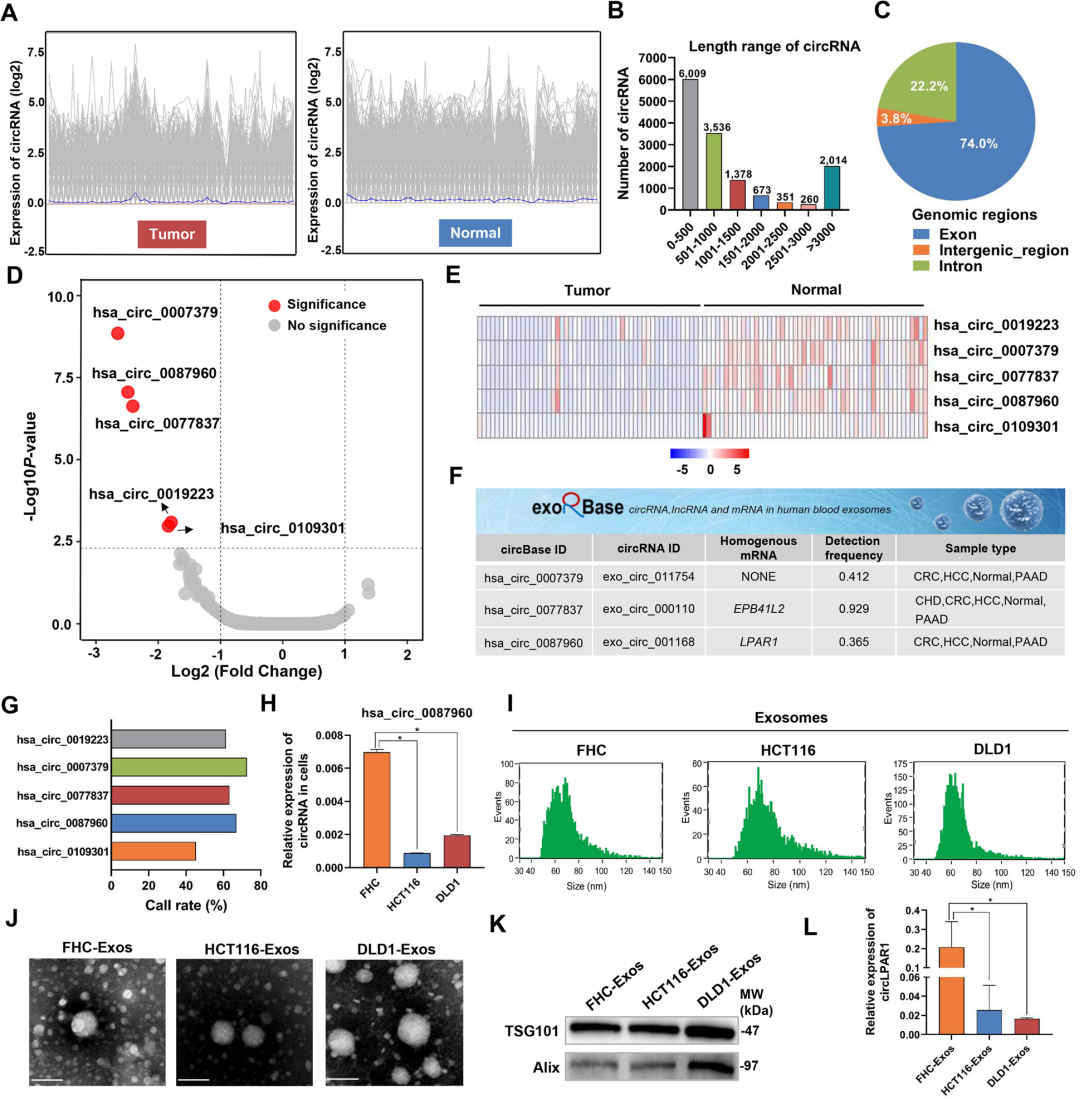
Profiling of circRNAs in tissues and selection of exosomal circLPAR1. The exosomes isolated from FHC, HCT116 and DLD1 cells were designated FHC-Exos, HCT116-Exos and DLD1-Exos, respectively. **A** The expression pattern of circRNAs in 52 pairs of colorectal tumors and normal adjacent tissues (NATs). The grey line shows the relative expression of circRNAs in each tissue (the log2 (ratios)). The blue line shows the average relative expression of all circRNAs in each tissue (the log2 (ratios)). The X-axis represents colorectal cancer tissues (left) and NATs (right). **B** Spliced length distributions (nt) of circRNAs as determined by RNA-Seq. **C** Pie chart showing the percentage of circRNAs derived from different genomic regions. **D** Volcano plots of differentially expressed circRNAs (fold-change (FC) ≥ |2| and *P* < 0.005). **E** The cluster heat map of five differentially expressed circRNAs. **F** The characteristics of differentially expressed circRNAs available in the exoRBase 1.0 database in 2018. **G** The call rate of differentially expressed circRNAs identified by RNA-Seq. **H** Differential expression of hsa_circ_0087960 between normal colorectal mucosal cells (FHC) and colorectal cancer cells (HCT116 and DLD1). Statistical significance was assessed using two-tailed Student’s *t*-test. **I** The size distributions of FHC/HCT116/DLD1-Exos. Exosomes were identified by NanoFCM. The X-axis represents exosome size and the Y-axis represents exosome number. **J** Visualization of purified FHC/HCT116/DLD1-Exos. TEM was performed to confirm the shape of the exosomes. Scale bar, 100 nm. **K** Western blot showing the exosome-specific markers TSG101 and Alix. **L** Differential expression of exosomal circLPAR1 between the FHC-Exos and HCT116/DLD1-Exos groups. Statistical significance was assessed using two-tailed Student’s *t*-test. The values represent mean ± SD.^*^P < 0.05

To characterize dysregulated circRNAs in colorectal cancer, we performed RNA-Seq in 52 pairs of colorectal tumors and NATs and observed 14,221 circRNAs (Fig. 1A and Table S1). Among these circRNAs, most were less than 500 nt (Fig. 1B), and more than 70% of these circRNAs originated from protein-coding exons, followed by introns and intergenic regions (Fig. 1C). After establishing the filter criteria, a fold-change (FC) ≥ |2| and *P* < 0.005, we obtained five circRNAs (hsa_circ_0019223, hsa_circ_0007379, hsa_circ_0077837, hsa_circ_0087960, and hsa_circ_0109301) with significantly decreased expression in colorectal tumor tissues compared with their expression in NATs (Fig. 1D-E and Table S2). Furthermore, we used the exoRBase database to annotate the circRNAs in exosomes and found three exosomal circRNAs (hsa_circ_0007379, hsa_circ_0077837 and hsa_circ_0087960; Fig. 1F) with high abundance, as determined by RNA-Seq (Fig. 1G). Notably, hsa_circ_0087960 (derived from *LPAR1*, hereafter termed circLPAR1) was the only circRNA to be validated in cell lines with significantly downregulated expression in colorectal cancer cells (HCT116 and DLD1) compared with normal FHC cells (Fig. 1H and Fig. S1A-B).

Subsequently, we purified exosomes from the cell culture medium (FHC-Exos and HCT116/DLD1-Exos) and observed typical exosome features: diameter ranging from 50 nm to 100 nm (Fig. 1I), a cup-shaped morphology (Fig. 1J), and expressed marker proteins TSG101 and Alix (Fig. 1K). Consistent with the expression pattern in parent cells, the expression of circLPAR1 was lower in HCT116/DLD1-Exos than in FHC-Exos (Fig. 1L). In addition, the circLPAR1 level in the cell culture medium was significantly decreased after treatment with the exosome secretion inhibitor GW4869 (Fig. S1C-D).

### CircLPAR1 is downregulated in colorectal tumors and associated with overall survival

**Fig. 2:**
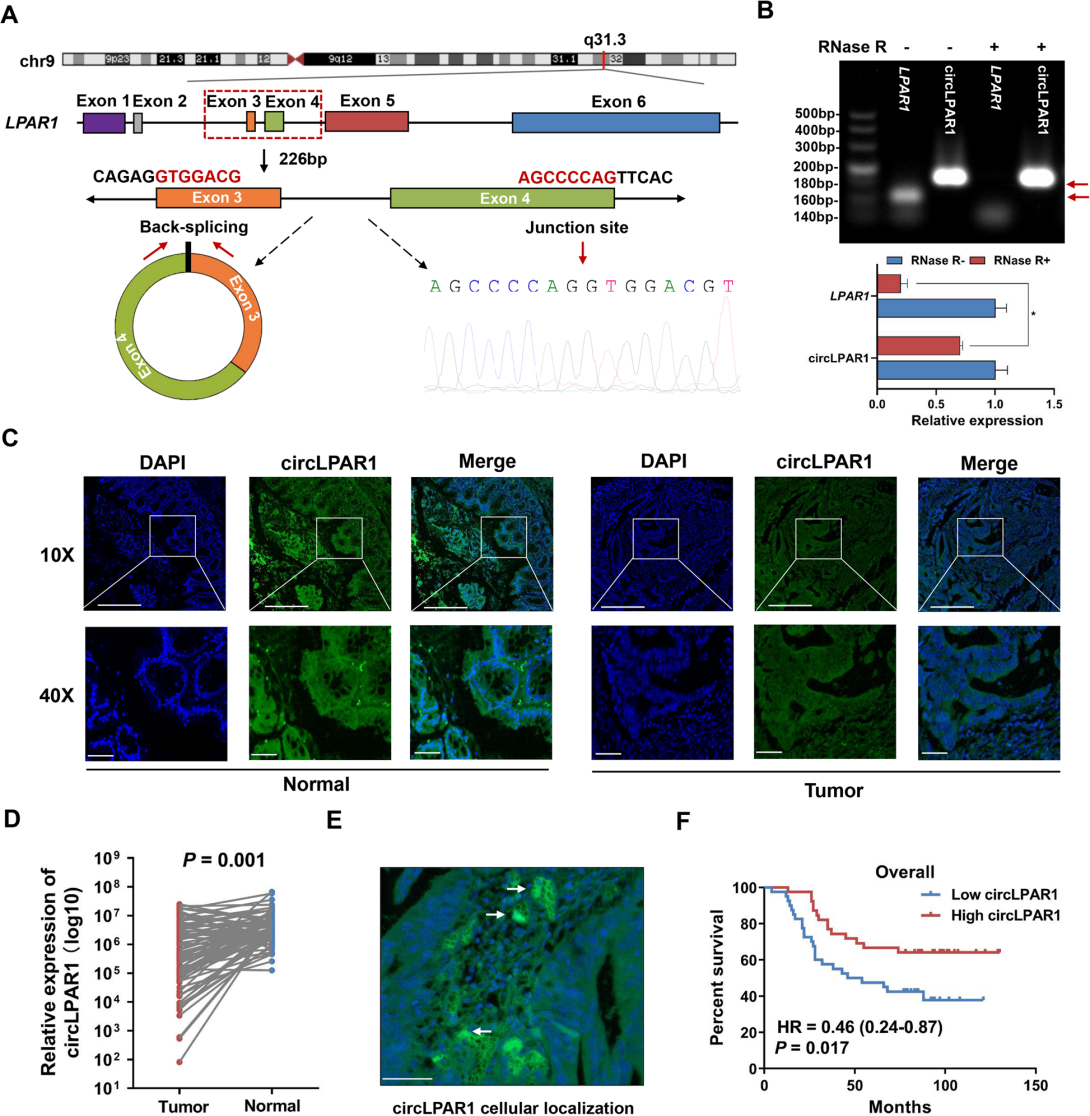
Characterization of circLPAR1 in colorectal cancer tissues. **A** Schematic illustration of circLPAR1 formation through the circularization of exons 3 and 4 in *LPAR1*. The back-splice junction sequences were confirmed by Sanger sequencing. **B** Relative expression of *LPAR1* mRNA and circLPAR1 after RNase R digestion. **C** Representative fluorescence images of circLPAR1 (green) in colorectal cancer tissues and NATs, as obtained by FISH. Scale bar, 250 μm (top) and 50 μm (bottom). **D** The relative expression of circLPAR1 in 79 pairs of colorectal cancer tissues and NATs measured by FISH. Statistical significance was assessed using two-tailed paired Student’s *t*-test. **E** The cellular localization of circLPAR1 in colorectal cancer tissue as determined by FISH. Scale bar, 50 μm; white arrowhead indicates circLPAR1 cellular localization. **F** The association of circLPAR1 expression level and overall survival in colorectal cancer patients. Kaplan-Meier analysis was used to plot and calculate the *P* value, and Cox regression was used to evaluate HR and 95% CI. ^*^P < 0.05

CircLPAR1 is generated from exon 3 and exon 4 circularization of the *LPAR1* gene located on chr9 (113734352-113735838) and is 226 bp long with a “head-to-tail” back-splice junction site, as evidenced by Sanger sequencing (Fig. 2A). To examine the stability of circLPAR1, we treated colorectal cancer cells with actinomycin D (an inhibitor of transcription) and observed that circLPAR1 was more stable than linear *LPAR1* transcripts (Fig. S2A). In addition, we found that circLPAR1 was resistant to RNase R digestion (a degrader of the linear RNA) compared with the linear *LPAR1* transcripts (Fig. 2B). To visualize the expression pattern of circLPAR1, we performed FISH assays with a tissue microarray containing 79 pairs of colorectal tumors and NATs (Table S3) and obtained findings that aligned with the aforementioned RNA-Seq data showing that circLPAR1 was significantly expressed at lower levels in colorectal tumors than in NATs (*P* = 0.001; Fig. 2C-D). Notably, circLPAR1 was predominantly expressed and localized in the cytoplasm (Fig. 2E). In addition, when colorectal cancer patients were stratified on the basis of the median expression of circLPAR1 (Fig. S2B), a Kaplan-Meier analysis of the data indicated that colorectal cancer patients with a high circLPAR1 level exhibited significantly better overall survival than those with a low circLPAR1 level (HR = 0.46, 95% confidence interval (CI) = 0.24–0.87; *P*_log-rank_ = 0.017; Fig. 2F).

### Plasma exosomal circLPAR1 is a specific biomarker for colorectal cancer diagnosis

**Fig. 3:**
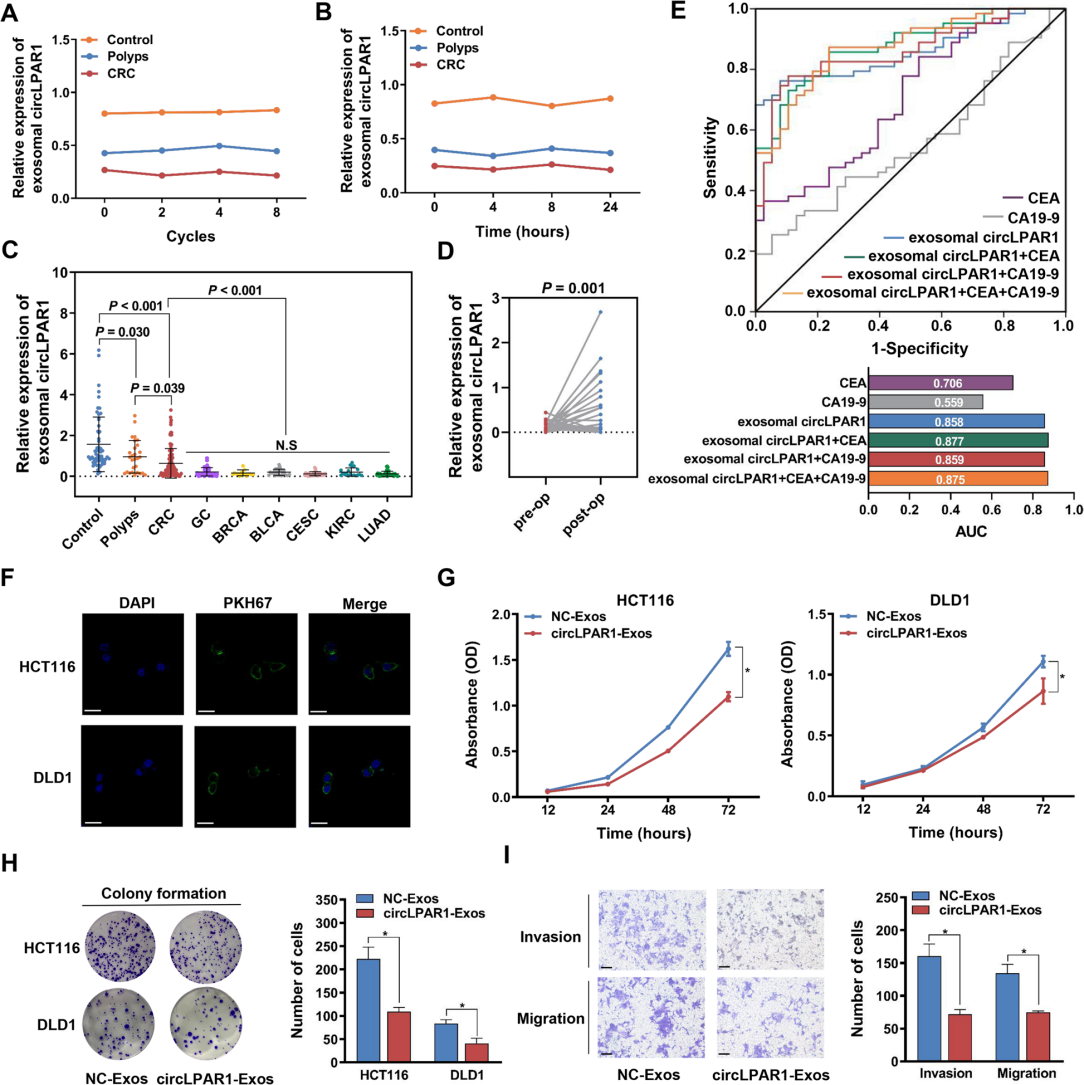
The expression pattern of plasma exosomal circLPAR1 and the biological effect of exosomal circLPAR1 on colorectal cancer cellular phenotypes. The exosomes isolated from HCT116 and DLD1 cells were designated HCT116-Exos and DLD1-Exos, respectively. Exosomes were isolated from HCT116 and DLD1 cells transfected with circLPAR1/controls expression plasmid, namely, circLPAR1-Exos and NC-Exos, respectively. **A** The expression of exosomal circLPAR1 was detected after thawing and freezing human plasma repeatedly for 0 cycles, 2 cycles, 4 cycles, and 8 cycles. **B** The expression of exosomal circLPAR1 was detected after placing human plasma at room temperature for 0 h, 4 h, 8 h, and 24 h. **C** The expression pattern of plasma exosomal circLPAR1 across cancer-free control individuals, and patients with polyps, colorectal cancer, or other types of cancer. **D** Altered expression of plasma exosomal circLPAR1 in colorectal cancer patients before and after operation (op). **E** Top, ROC curves for CEA, CA19–9 and exosomal circLPAR1 alone or in combination. Bottom, the AUC values for CEA, CA19–9, and exosomal circLPAR1 alone or in combination. **F** Representative fluorescence images of colorectal cancer cells after incubation with HCT116/DLD1-Exos labelled with PKH67 (green). Scale bar, 25 μm. **G** The proliferation of HCT116 and DLD1 cells measured by CCK-8 assay (OD450 absorbance) after incubation with circLPAR1/NC-Exos. **H** The colony formation activity of HCT116 and DLD1 cells evaluated by colony formation assay after incubation with circLPAR1/NC-Exos. **I** The invasion and migration ability of DLD1 cells after incubation with circLPAR1/NC-Exos as detected by a transwell assay. Scale bar, 100 μm. Statistical significance was assessed using two-tailed Student’s *t*-test. The values represent mean ± SD. ^*^P < 0.05, N. S, not significant

Considering the detection of circLPAR1 in exosomes and its downregulated expression pattern in colorectal tumors, we hypothesized that circLPAR1 is carried by plasma exosomes and acts as a specific biomarker for colorectal cancer. We successfully identified plasma exosomes by analysing their morphology, marker expression and size (Fig. S3). Furthermore, we observed that exosomal circLPAR1 level in plasma did not obviously change (*P* > 0.05) after subjection to different freeze-thaw cycles (Fig. 3A) or maintenance at room temperature for different times (Fig. 3B) across each divided sample. Notably, exosomal circLPAR1 was significantly lower in colorectal cancer patients than in cancer-free controls (*P* < 0.001; Fig. 3C and Table S4) and patients with polyps (*P* = 0.039; Fig. 3C and Table S5), indicating that exosomal circLPAR1 may be decreased during disease development from precancer to cancer. Interestingly, we found similar expression patterns of exosomal circLPAR1 in many cancers, including GC, BRCA, BLCA, CESC, KIRC, and LUAD (*P* > 0.05; Fig. 3C and Table S5), and these patterns were all notably different from the pattern in colorectal cancer (*P* < 0.001; Fig. 3C). Moreover, the exosomal circLPAR1 level was significantly increased after colorectal tumor resection (*P* = 0.001; Fig. 3D and Table S6). As shown in Fig. 3E, the ROC curve revealed that exosomal circLPAR1 can be measured to distinguish colorectal cancer patients from cancer-free control individuals with AUC of 0.858 (95% CI = 0.786–0.930, *P <* 0.001). CEA and CA19–9 are two widely used clinical biomarkers for colorectal cancer diagnosis that were significantly upregulated in patient plasma (Fig. S4) but had a relatively low AUC (CEA: AUC = 0.706; CA19–9: AUC = 0.559; Fig. 3E and Fig. S5) compared with exosomal circLPAR1. Notably, the combination of exosomal circLPAR1, CEA and CA19–9 increased the AUC value up to 0.875 (95% CI = 0.809–0.941, *P <* 0.001; Fig. 3E), accompanied by 87.30 and 76.30% sensitivity and specificity, respectively.

### Exosomal circLPAR1 is transmitted among cells and inhibits cellular phenotypes

Next, we explored the biological function of exosomal circLPAR1 in colorectal tumorigenesis. Fluorescence microscopy showed PKH67 green fluorescent dye in the cytoplasm of HCT116 and DLD1 cells, while no PKH67 green fluorescent dye was observed in the non-Exos group, suggesting that HCT116/DLD1-Exos were rapidly engulfed by colorectal cancer cells (Fig. 3F and Fig. S6); however, this uptake was eliminated by the inhibition of endocytosis with cytochalasin D (Fig. S7). Subsequently, we isolated exosomes from colorectal cancer cells that were transfected with the circLPAR1/NC vector (circLPAR1/NC-Exos) and found that direct incubation with circLPAR1-Exos significantly increased circLPAR1 levels in HCT116 and DLD1 cells (Fig. S8). Intriguingly, we observed that exosomal circLPAR1 dramatically suppressed the proliferation, colony formation, invasion, and migration of colorectal cancer cells (Fig. 3G-I and Fig. S9), a finding similar to that obtained by the direct overexpression of circLPAR1 (Fig. S10).

### Exosomal circLPAR1 suppresses colorectal cancer tumorigenesis by regulating *BRD4* levels upon interaction with eIF3h

**Fig. 4:**
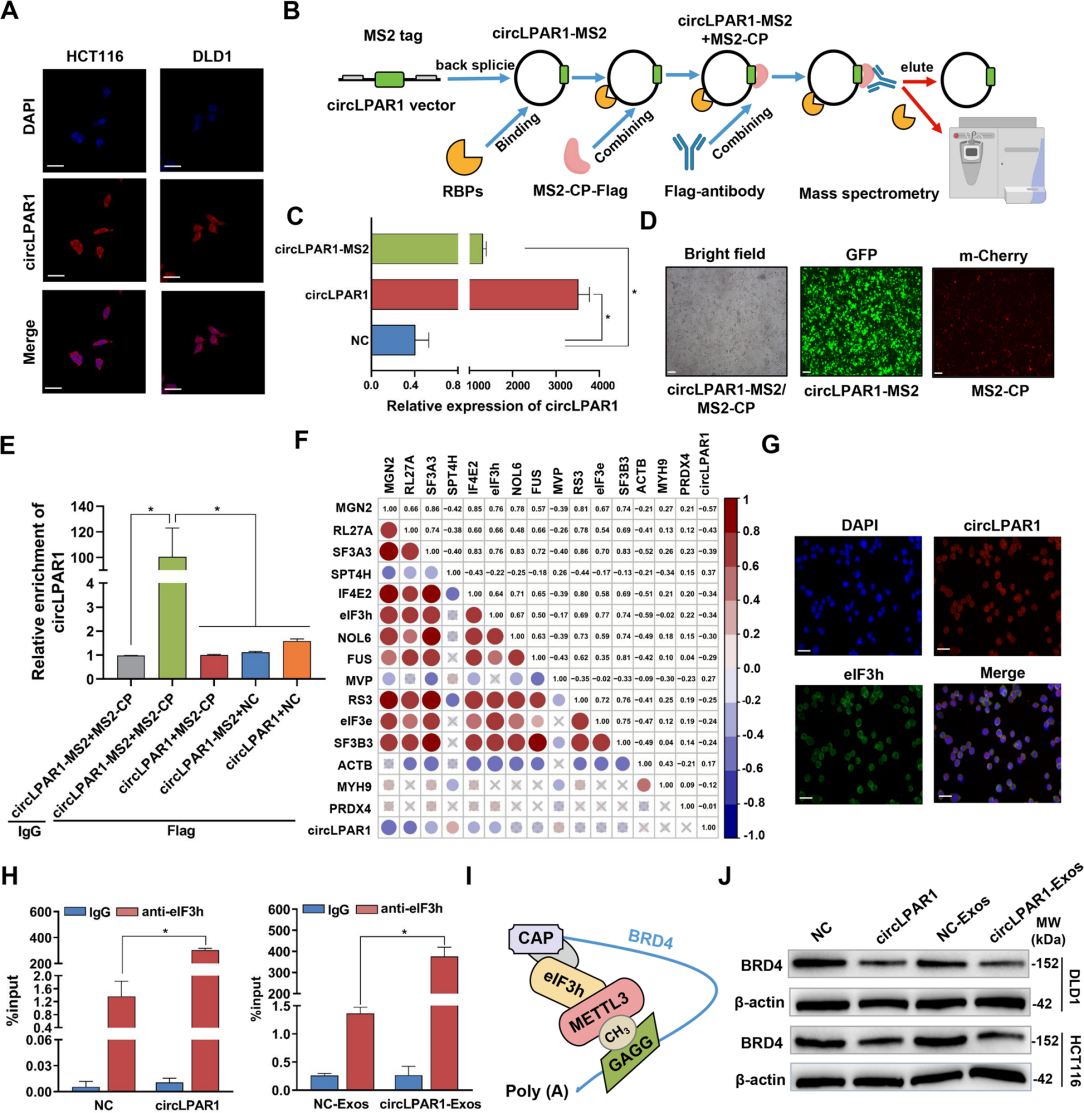
Exosomal circLPAR1 regulates the translation of *BRD4* by binding to eIF3h. The circLPAR1 and control lentiviral vectors were transfected separately into DLD1 cells and used as the circLPAR1 group and NC group, respectively. Exosomes were isolated from DLD1 cells transfected with circLPAR1 and control lentiviral vector separately and used as the circLPAR1-Exos and NC-Exos group, respectively. **A** The cellular location of circLPAR1 (red) in HCT116 (left) and DLD1 (right) cells as determined by FISH. Scale bar, 25 μm. Nuclei were stained with DAPI (blue). **B** Flow chart showing circLPAR1 pull-down results using the MS2-tagging system. **C** HCT116 cells were transfected separately with circLPAR1, control or MS2-labelled circLPAR1 expression plasmids and called circLPAR1, NC and circLPAR1-MS2, respectively. The relative expression of circLPAR1 as detected by RT-qPCR in NC, circLPAR1 and circLPAR1-MS2 cells. **D** Co-transfection of circLPAR1-MS2 and MS2-CP into HCT116 cells. Representative fluorescence images of circLPAR1-MS2 (green) and MS2-CP (red). Scale bar, 50 μm. **E** The enrichment of circLPAR1 in complex with MS2-CP-Flag as measured by RT-qPCR. **F** Correlation between the level of circLPAR1 and 15 differentially expressed proteins based on 25 pairs of colorectal cancer tissues and NATs. **G** Immunofluorescence assessment of circLPAR1 (red) and eIF3h (green) colocalization in DLD1 cells. Scale bar, 20 μm. **H** RIP assay with an anti-eIF3h antibody or control IgG performed on circLPAR1/NC cells (left) or circLPAR1/NC-Exos (right). The expression of circLPAR1 in the immunoprecipitates was detected using RT-qPCR. **I** Model of the interaction between eIF3h and METTL3 to regulate translation of *BRD4*. (J) The BRD4 protein in circLPAR1/NC cells or circLPAR1/NC-Exos as determined by Western blotting. Statistical significance was assessed using two-tailed Student’s *t*-test. The values represent mean ± SD. *P < 0.05

CircLPAR1 was enriched in the cytoplasm in both cell lines and tissues (Fig. 2E, Fig. 4A, and Fig. S11), prompting us to profile the specific circRNA-RBP complex influenced by circLPAR1. We performed proteomic profiling by MS2-CP-Flag circRNA pull-down assay (Fig. 4B). As shown in Fig. 4C-D, the transfection efficiency of circLPAR1-MS2 vector or MS2-CP was confirmed. The circRNA pull-down products were enriched with the capture protein MS2-CP-Flag (Fig. S12), and circLPAR1 was highly abundant in the following capture (Fig. 4E); both experiments indicate the successful specificity of the circRNA pull-down assay. The following mass spectrometry analysis led to the identification of 23 candidate RBPs assigned to circLPAR1 via comparison between the circLPAR1 + MS2-CP-transfected group and circLPAR1-MS2 + MS2-CP-transfected group (Table S7). We further examined the differential protein expression of these RBPs based on 25 pairs of colorectal tumors and NATs (Table S1) and found that 12 RBPs were upregulated and three RBPs were downregulated in tumors (Fig. S13). In addition, circLPAR1 was significantly correlated with six RBPs (MGN2, RL27A, SF3A3, SPT4H, IF4E2, and eIF3h; *P* < 0.05; Fig. 4F), among which four RBPs (MGN2, RL27A, SF3A3, and eIF3h) were positively associated with their parental linear RNAs (Table S8). Furthermore, we estimated the binding capacity of these four RBPs specifically for circLPAR1 with the catRAPID signature/express module and found that eIF3h had the strongest binding capacity with circLPAR1 (score = 0.68, interaction propensity = 24 and discrimination power = 64%; Table S8). Therefore, we focused the downstream study on eIF3h, and the spectrum of eIF3h by mass spectrometry analysis is shown in Fig. S14.

To confirm the binding affinity of circLPAR1 for eIF3h, we first observed them and identified the colocalization of circLPAR1 and eIF3h in the cytoplasm of colorectal cancer cells (Fig. 4G). Moreover, a RIP assay verified that circLPAR1 or exosomal circLPAR1 was specifically precipitated with eIF3h from colorectal cancer cells transfected with a circLPAR1 lentivirus vector or incubated with circLPAR1-Exos, in contrast to the NC/NC-Exos group cells (Fig. 4H), indicating that exosomal circLPAR1 directly interacts with eIF3h. A recent study reported that there is a direct physical and functional interaction between eIF3h and methyltransferase-like 3 (METTL3) [34], which can regulate bromodomain-containing protein 4 (*BRD4*) translation and influence oncogenesis (Fig. 4I). Accordingly, we wondered whether exosomal circLPAR1 can serve as a sponge of eIF3h to influence BRD4 protein expression. As shown in Fig. 4J, circLPAR1 reduced BRD4 protein levels in colorectal cancer cells transfected with circLPAR1 lentiviral vector or incubated with circLPAR1-Exos compared with the NC/NC-Exos group. To exclude the regulating effect of circLPAR1 on *BRD4* levels, we transfected a *BRD4* expression vector into stable circLPAR1-expressing cells, which were then treated with the specific *BRD4* inhibitor AZD5153. The *BRD4* expression plasmid elevated the decrease in *BRD4* levels caused by circLPAR1 expression and promoted the acquisition of colorectal cancer cell phenotypes, whereas AZD5153 reversed the tumor-promoting effect of high *BRD4* levels (Fig. S15). It has been reported that circLPAR1 can sponge miR-762 to promote the invasion and metastasis of bladder cancer [35]. We therefore measured the miR-762 level in the aforementioned circRNA pull-down products but found no enrichment of miR-762 with circLPAR1 (Fig. S16).

### Exosomal circLPAR1 suppresses colorectal cancer growth in vivo

**Fig. 5:**
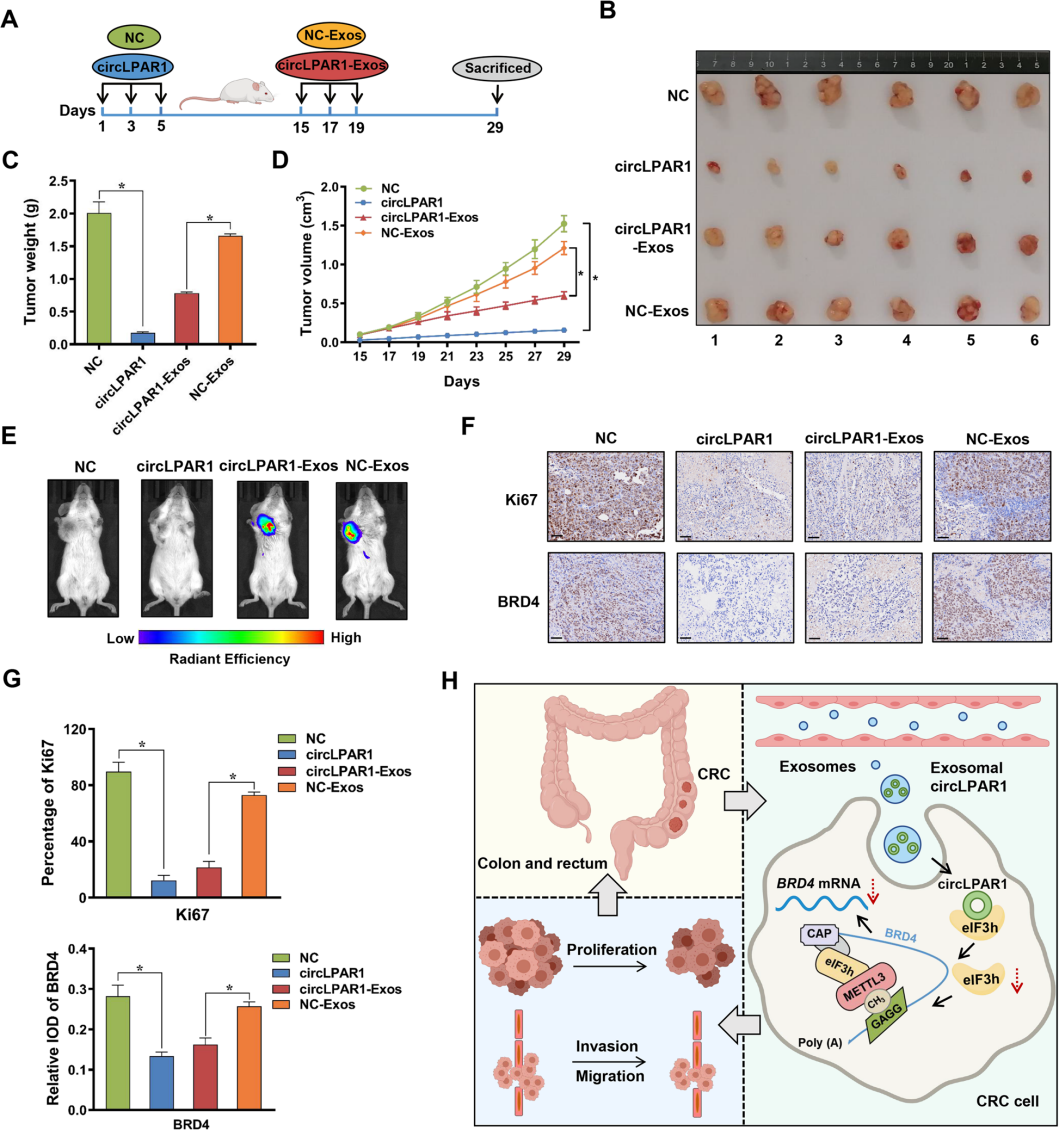
The effect of targeting exosomal cricLPAR1 in vivo. DLD1 cell lines transfected with circLPAR1/NC lentiviral vector were injected into humanized NCG mice, which were designated circLPAR1 and NC, and then DiR-labelled circLPAR1/NC-Exos were injected into NC mice, which were designated circLPAR1-Exos and NC-Exos. **A** Schematic diagram showing the process of establishing the mouse model of exosomal circLPAR1. **B** Harvested tumor tissues in the NC, circLPAR1, circLPAR1-Exos, and NC-Exos groups. The average tumor weight (**C**) and the mean tumor volumes (**D**) in the NC, circLPAR1, circLPAR1-Exos, and NC-Exos groups. **E** Representative images of mice after injection of DiR-labelled exosomes in the NC, circLPAR1, circLPAR1-Exos, and NC-Exos groups. Luminescence intensity ranges from low (blue) to high (red). **F** Representative images of Ki67 and BRD4 immunohistochemistry results. Scale bar, 50 μm. **G** The levels of Ki67 and BRD4 in immunohistochemistry staining. **H** Schematic diagram illustrating that exosomal circLPAR1 acted as a sponge of eIF3h to reduce the METTL3-eIF3h interaction, which inhibited the translation of *BRD4*, inducing suppression of colorectal cancer tumorigenesis. Statistical significance was assessed using two-tailed Student’s *t*-test. The values represent mean ± SD. ^*^P < 0.05

To validate the inhibition of exosomal circLPAR1 in colorectal cancer in vivo, we injected stable circLPAR1/NC-overexpressing cells or 1,1-dioctadecyl-3,3,3,3-tetramethylindotricarbocyanine iodide (DiR)-stained circLPAR1/NC-Exos into humanized NCG mice (Fig. 5A). Consistent with the in vitro results, compared with NC expression or NC-Exos treatment, circLPAR1 overexpression or circLPAR1-Exos treatment reduced the average size and weight of tumors (Fig. 5B-C). Moreover, the tumor tissues derived from the circLPAR1 and circLPAR1-Exos groups exhibited slower growth than those derived from the NC/NC-Exos group (Fig. 5D). The results of live imaging also showed that DiR-stained circLPAR1/NC-Exos were successfully injected into mice (Fig. 5E). Tumors in the circLPAR1 or circLPAR1-Exos group presented with higher levels of circLPAR1 than those in the NC/NC-Exos group (Fig. S17A). In addition, haematoxylin and eosin (H&E) staining and immunohistochemical (IHC) analyses showed impaired Ki67 and BRD4 in the circLPAR1 and circLPAR1-Exos group (Fig. 5F-G and Fig. S17B).

## Discussion

In this study, we identified a novel circRNA, circLPAR1, stably carried in plasma exosomes, and it emerged as a potential diagnostic biomarker specific for colorectal cancer. Mechanistically, exosomal circLPAR1 is internalized by colorectal cancer cells and binds eIF3h to reduce METTL3-eIF3h-dependent mRNA translation, thereby inhibiting *BRD4* expression and suppressing cellular proliferation, invasion, and migration (Fig. 5H).

Circular lysophosphatidic acid receptor 1 (circLPAR1, hsa_circ_0087960), has rarely been reported to be in tumors; however, one study on muscle-invasive bladder cancer showed that a decreased circLPAR1 level in bladder tumors was associated with bladder cancer prognosis [35]. Similarly, according to the evidence we obtained from circRNA expression profiling and a FISH analysis, circLPAR1 was expressed at low levels in colorectal cancer tissues and that its lower expression was associated with poor survival in patients with colorectal cancer. These observations suggest that circLPAR1 may be a circRNA specific to colorectal cancer. Notably, circLPAR1 was significantly lower in plasma exosomes obtained from patients with bladder cancer than in those from cancer-free control individuals, which is consistent with the report of Lin et al. [35].

The advantages of exosomal circLPAR1 as a biomarker are based on three aspects. First, circLPAR1 was resistant to actinomycin D and RNase R, indicating that it is more stable than its cognate linear *LPAR1* mRNA; circLPAR1 was encapsulated in exosomes, which conferred even greater protection against degradation. These characteristics rendered circLPAR1 exceptionally stable in plasma exosomes in multiple samples, such as the samples obtained from colorectal cancer, precancer, or cancer-free control individuals. Second, plasma exosomal circLPAR1 could distinguish colorectal cancer patients from cancer-free control individuals with acceptable sensitivity and specificity. Intriguingly, the plasma exosomal circLPAR1 level in colorectal cancer patients was significantly higher than that in patients with other types of cancer. Third, exosomal circLPAR1 significantly suppressed colorectal cancer cellular phenotypes, which is helpful both for exploring the biological mechanism of colorectal cancer and for offering new therapeutic approaches. These results collectively support the supposition that exosomal circLPAR1 can be used as a specific biomarker for colorectal cancer diagnosis.

An increasing number of studies have revealed that exosomes derived from certain parent cells can reach corresponding recipient cells in which they release their cargos to influence biological functions and induce a series of phenotypic changes [36, 37]. In this study, we found that circLPAR1 expression was dramatically elevated in exosomes isolated from colorectal cancer cell lines transfected with circLPAR1-expressing lentivirus or vector. In contrast, treatment with GW4869, a noncompetitive, specific, potent inhibitor of membrane neutral sphingomyelinase (nSMase) that blocks exosome production [38], resulted in significantly reduced expression of circLPAR1 in cell culture medium. Moreover, we noticed that transfection of circLPAR1 overexpression vectors into parent cells promoted the capacity of exosomal circLPAR1 to inhibit the acquisition of colorectal cancer malignant phenotypes, which was consistent with the effect of the direct overexpression of circLPAR1. In addition, circLPAR1 expression was much higher in the tumor tissues of circLPAR1-Exos xenograft-bearing mice than in NC-Exos group. These observations indicate that extracellular circLPAR1 can inhibit the acquisition of multiple malignant phenotypes of colorectal cancer when it is incorporated into exosomes.

The eIF3h was reported as one of the subunits of the eIF3 complex, which moderates several steps of initiation and elongation during protein synthesis [39]. Evaluating the MS2-CP circRNA pull-down assay, mass spectrometry analysis and proteomic profiling validation, we found that eIF3h directly binds to circLPAR1 and negatively correlates with circLPAR1. Notably, previous studies have shown that METTL3 can interact with the eIF3h subunit at the 5′-end of *BRD4* mRNA to enhance translation and promote oncogenesis [34]. *BRD4*, a member of the bromodomain and extra-terminal domain (BET) family, plays a crucial role in the development of multiple cancers and is regarded as a novel cancer therapeutic target [40, 41]. In addition, *BRD4* has been shown to promote the proliferation of colorectal cancer cells, while the *BRD4* inhibitor AZD5153 reversed this effect [42]. Our results extended the findings showing that exosomal circLPAR1 binds eIF3h to inhibit the METTL3–eIF3h interaction, which decreases the translation of *BRD4*, thereby suppressing colorectal cancer cell proliferation, invasion and migration, and experiments with in vivo models showed consistent results. Although Lin et al. found that circLPAR1 could bind to miR-762 and moderated the invasion of bladder cancer cells [35], we found that miR-762 was not enriched with circLPAR1 pull-down products. This inconsistent finding might be a result of different molecular mechanisms of circLPAR1 action in different types of cancer. In addition, more flexible screening criteria will be used in further RNA-Seq analyses, such as survival analysis or functional analysis, to obtain more effective circRNAs.

In summary, this study highlighted a novel colorectal cancer-associated circRNA, termed exosomal circLPAR1, which was easily and reliably detected in exosomes obtained from plasma and might act as a promising noninvasive biomarker for population screening and early detection of colorectal cancer. Mechanistically, exosomal circLPAR1 expression level led to decreased *BRD4* levels because it binds eIF3h and inhibits the METTL3–eIF3h interaction, which remarkably suppressed colorectal cancer development. This study not only reports a novel mechanism of exosomal circRNA in colorectal cancer, but also opens a new avenue for screening strategies and diagnostic approaches to colorectal cancer.

## Supplementary Information

**Additional file 1:** **Supplementary Materials and Methods. Table S1.** Demographic and clinical characteristics of the subjects who participated in RNA-Seq and proteogenomic analysis. **Table S2.** Five differentially expressed circRNAs and significant fold changes. **Table S3.** Demographic and clinical characteristics of the subjects in FISH analysis. **Table S4.** Demographic and clinical characteristics of subjects with colorectal cancer and cancer-free controls. **Table S5.** Characteristics of patients with other types of cancer. **Table S6.** Demographic and clinical characteristics of the colorectal cancer patients before and after operation. **Table S7.** Characterization of circLPAR1-binding proteins. **Table S8.** The correlation between protein and its parental gene expression and prediction of RNA binding ability. **Table S9.** Sequences of circLPAR1 used for FISH analysis in the study. **Table S10.** Sequences of primers used for RT-qPCR in the study. **Figure S1.** The expression of circRNAs detected by RT-qPCR. **Figure S2**. The expression of circLPAR1 in CRC cells treated with actinomycin D and colorectal tumors. **Figure S3.** Characterization of exosomes derived from human plasma. **Figure S4.** The expression of traditional clinical biomarkers in plasma. **Figure S5**. ROC curve analysis of exosomal circLPAR1 and traditional clinical biomarkers in human plasma. **Figure S6.** Fluorescence observation of colorectal cancer cells after incubation with PKH67-labelled exosomes or non-exosomes. **Figure S7.** Fluorescence observation of colorectal cancer cells incubation with PKH67-labelled exosomes pretreated with cytochalasin D. **Figure S8**. The expression of circLPAR1 in exosomes derived from colorectal cancer cells. **Figure S9**. The effect of exosomal circLPAR1 on invasion and migration abilities of HCT116 cells. **Figure S10**. The effect of circLPAR1 overexpression on colorectal cancer cellular phenotypes. **Figure S11.** The cellular localization of circLPAR1 in colorectal cancer cells. **Figure S12**. MS2-CP-Flag was pulled down by anti-flag and evaluated by Western blot. **Figure S13.** The proteomic analysis showing 23 proteins levels based on 25 paired colorectal cancer tissues. **Figure S14**. Mass spectrometry analysis identification of eIF3h. **Figure S15**. The effect of *BRD4* regulation by circLPAR1 on colorectal cancer cellular phenotype. **Figure S16**. The enrichment of miR-762 in the complex with MS2-CP-Flag. **Figure S17**. Effect of exosomal circLPAR1 on colorectal cancer progression in vivo.

## Data Availability

Not applicable.

## Abbreviations

CircRNAs: Circular RNAs
CRC: Colorectal cancer
Exos: Exosomes
RNA-Seq: RNA sequencing
GC: Gastric carcinoma
BRCA: Breast invasive carcinoma
BLCA: Bladder urothelial carcinoma
CESC: Cervical squamous cell carcinoma and endocervical adenocarcinoma
KIRC: Kidney renal clear cell carcinoma
LUAD: Lung adenocarcinoma
NATs: Normal adjacent tissues
FISH: Fluorescence in situ hybridization
eIF3h: Eukaryotic translation initiation factor 3 subunit h
TEM: Transmission electron microscopy
RBPs: RNA-binding proteins
MS2-CP: MS2-capturing protein
RT-qPCR: Real-time quantitative polymerase chain reaction
RIP: RNA immunoprecipitation
ROC: The receiver operating characteristic
AUC: Area under the curve
CircLPAR1: Circular lysophosphatidic acid receptor 1
METTL3: Methyltransferase-like 3
*BRD4*: Bromodomain-containing protein 4
