# Supplementary material for: Exosomal circLPAR1 functions in colorectal cancer diagnosis and tumorigenesis through suppressing BRD4 via METTL3–eIF3h interaction
Source: Mol Cancer. 2022 Feb 14;21:49. doi: 10.1186/s12943-021-01471-y (PMC8842935; doi:10.1186/s12943-021-01471-y)

**Supplementary Materials and Methods**

**Supplementary Tables**

**Table S1.** Demographic and clinical characteristics of the subjects who participated in RNA-Seq and proteogenomic analysis

**Table S2.** Five differentially expressed circRNAs and significant fold changes

**Table S3.** Demographic and clinical characteristics of the subjects in FISH analysis

**Table S4.** Demographic and clinical characteristics of subjects with colorectal cancer and cancer-free controls

**Table S5.** Characteristics of patients with other types of cancer

**Table S6.** Demographic and clinical characteristics of the colorectal cancer patients before and after operation

**Table S7.** Characterization of circLPAR1-binding proteins

**Table S8.** The correlation between protein and its parental gene expression and prediction of RNA binding ability

**Table S9.** Sequences of circLPAR1 used for FISH analysis in the study

**Table S10.** Sequences of primers used for RT-qPCR in the study

**Supplementary Figures**

Fig. S1. The expression of circRNAs detected by RT-qPCR.

Fig. S2. The expression of circLPAR1 in CRC cells treated with actinomycin D and colorectal tumors.

Fig. S3. Characterization of exosomes derived from human plasma.

Fig. S4. The expression of traditional clinical biomarkers in plasma.

Fig. S5. ROC curve analysis of exosomal circLPAR1 and traditional clinical biomarkers in human plasma.

Fig. S6. Fluorescence observation of colorectal cancer cells after incubation with PKH67-labelled exosomes or non-exosomes.

Fig. S7. Fluorescence observation of colorectal cancer cells incubation with PKH67-labelled exosomes pretreated with cytochalasin D.

Fig. S8. The expression of circLPAR1 in exosomes derived from colorectal cancer cells.

Fig. S9. The effect of exosomal circLPAR1 on invasion and migration abilities of HCT116 cells.

Fig. S10. The effect of circLPAR1 overexpression on colorectal cancer cellular phenotypes.

Fig. S11. The cellular localization of circLPAR1 in colorectal cancer cells.

Fig. S12. MS2-CP-Flag was pulled down by anti-flag and evaluated by Western blot.

Fig. S13. The proteomic analysis showing 23 proteins levels based on 25 paired colorectal cancer tissues.

Fig. S14. Mass spectrometry analysis identification of eIF3h.

Fig. S15. The effect of *BRD4* regulation by circLPAR1 on colorectal cancer cellular phenotype.

Fig. S16. The enrichment of miR-762 in the complex with MS2-CP-Flag.

Fig. S17. Effect of exosomal circLPAR1 on colorectal cancer progression *in vivo*.

**Supplementary Materials and Methods**

**Cell lines and cell culture**

Human colorectal cancer cell lines (HCT116 and DLD1) and a normal colonic mucosal cell line (FHC) were purchased from Shanghai Institute of Cell Biology of Chinese Academy of Science (Shanghai, China). DLD1 and FHC cells were cultured with RPMI 1640 medium (Biological Industries, USA); HCT116 cells were cultured in McCoy’s 5A medium (Biological Industries, USA), containing 10% heat-inactivated foetal bovine serum (FBS) (Biological Industries, USA), 100 μg/ml streptomycin (Gibco, USA) and 100 U/ml penicillin (Gibco, USA). The indicated cells used for exosome isolation were cultured with exosome-depleted FBS (SBI, USA). All cells were grown in a humidified incubator of 5% CO_2_ at 37°C.

**Total RNA and exosomal RNA extraction**

Total RNA was isolated from tissues or cell lines with TRIzol reagent (Thermo Fisher Scientific, USA), and exosomal circRNAs were extracted from plasma or culture medium of parent cells with an exoRNeasy Serum/Plasma Maxi kit (Qiagen, Germany) following the manufacturer’s instructions. The quantity and quality of isolated RNA were evaluated by using a Nanodrop 2000 spectrophotometer (Thermo Fisher Scientific, USA).

**Reverse transcription and RT-qPCR**

The cDNA was synthesized from circRNAs by the PrimeScript RT reagent kit with gDNA Eraser kit (TaKaRa, Japan) under the recommended conditions, cDNA was synthesized from mRNA with a high-capacity cDNA reverse transcription kit (Invitrogen, USA), and cDNA was synthesized from microRNAs (miRNAs) with a Reverse Transcriptase XL kit (AMV) (TaKaRa, Japan), dNTP Mixture (TaKaRa, Japan) and recombination RNase inhibitor (TaKaRa, Japan). RT-qPCR was performed with circRNAs, genes or miRNAs using a SYBR Green RT-PCR kit (Vazyme Biotech, China) on a Roche Light Cycler 480 II system (Roche, Switzerland). GAPDH was used as the internal control for circRNAs and mRNA, and U6 was used for the miRNAs. The relative expression levels of circRNAs, mRNAs or miRNAs were calculated by the 2^-∆Ct^ method. The sequences of specific primers or probe applied in this study are listed in **Table S9-10**.

**Vector construction and cell transfection**

To overexpress circLPAR1, the circLPAR1 cDNA was enlarged and then cloned into an pLC5-circR expression vector (Geneseed, China), and pLC5 vector was used as the control. The *BRD4* overexpression vector was constructed by GeneBay Biotech Co., Ltd. (China). All vectors were determined by sequencing. The colorectal cancer cells were transiently transfected with circLPAR1- or *BRD4*-overexpression plasmids using Lipofectamine 3000 (Invitrogen, USA) in accordance with the manufacturer’s instruction. Moreover, a lentiviral vector expressing circLPAR1 was constructed using the pLC5 infection system (GeneChem, China). In addition, AZD5153 (Selleck, China) was used to inhibit *BRD4* expression in colorectal cancer cells expressing circLPAR1 lentivirus.

**Exosome treatment**

The exosome concentration was measured using a bicinchoninic acid (BCA) protein assay kit (Beyotime Institute of Biotechnology, China). For the *in vitro* exosome incubation experiments, 2 µg of exosomes (equivalent to those isolated from ~1×10^7^ of the indicated producer cells) was incubated with 2×10^5^ colorectal cancer cells for 24 h. To inhibit exosome secretion, the indicated producer cells were pretreated with 20 μM GW4869 (Sigma, USA) or 0.005% DMSO (Sigma, USA) as a control, for 6 h, and the culture medium of the producer cells was prepared for RNA extraction.

**Western blot analysis**

The exosomal or indicated cell lysates were acquired with RIPA lysis buffer mixed with 0.5% phenylmethylsulfonyl fluoride (PMSF), and the concentration of protein was then measured using a BCA protein assay. Equal protein samples (60 μg) were resolved by sodium dodecyl sulfate–polyacrylamide gel electrophoresis (SDS-PAGE) and transferred to polyvinylidene fluoride (PVDF) membranes (Millipore, USA). After blocking, membranes were immunoblotted overnight at 4 °C with anti-BRD4 (# ab128874, Abcam, USA). β-Actin (# ab8226, Abcam, USA) was used as loading controls for normalization. Immunoreactive proteins were detected using a Bio-Rad gel imaging system (Bio-Rad, USA).

**Cell proliferation and colony formation assays**

The indicated cells (n = 3,000) were plated in 96-well plates and then incubated at 37°C for 12 h, 24 h, 48 h or 72 h. The proliferation activity of colorectal cancer cells was determined using a Cell Counting Kit-8 (Dojindo, Japan) following the manufacturer’s protocols. The absorbance of indicated cells was tested at 450 nm with an Infinite M200 spectrophotometer (Tecan, Switzerland). Colony-formation assays were carried out to evaluate the cloning capability of colorectal cancer cells. In brief, indicated cells were seeded onto 6-well culture plates until visible colonies appeared, and the complete medium was replaced every 4 days. Subsequently, the cell colonies were fixed in 95% methanol for 20 min, stained with 0.1% crystal violet (Beyotime, China) for 15 min and quantified using Image J software.

**Cell invasion and migration assays**

To determine the effects of exosomal circLPAR1, circLPAR1 or *BRD4* on colorectal cancer cells, Matrigel (Corning, USA) was first added to the top chamber of 8-μm transwell filters (Corning, USA). The next day, the indicated cells were plated into the upper chamber with 100 μl of serum-free medium, and complete medium was added to the lower chamber. After incubation for 24 h at 37°C, the cells in the upper chamber were removed, and the invaded cells were fixed with 95% methanol for 20 min and stained with 0.1% crystal violet for 15 min. Similar to the invasion assay, the migration assay was performed with transwell inserts but without Matrigel. The migrated cells were obtained and quantified following the processes described for the invasion assay.

**Nucleocytoplasmic separation**

The cytosolic and nuclear fractions of HCT116 and DLD1 cells were separated using a PARIS^TM^ kit according to the manufacturer’s instruction (Thermo Fisher Scientific, USA). The expression of circLPAR1, *GAPDH* and *U6* in the nucleus or cytoplasm of colorectal cancer cells was measured by RT-qPCR.

**Carcinoembryonic antigen (CEA) and CA19-9 assays**

The plasma levels of CEA and CA19-9 was detected using a human carcinoembryonic antigen (CEA) ELISA Kit (Cusabio, China) and a human carbohydrate antigen 19-9 (CA19-9) ELISA kit (Cusabio, China).

**Exosome tracking *in vivo***

To monitor whether exosomes entered the animal model, exosomes isolated from DLD1 cells stably transfected with circLPAR1/NC were labelled with the fluorescent lipophilic tracer 1,1-dioctadecyl-3,3,3,3-tetramethylindotricarbocyanine iodide (DiR; Meilun Biotechnology Co., Ltd., China). A total of 10 μg of exosomes were incubated with DiR at a concentration of 2.5 mg/ml PBS for 5 min at 37°C, incubated for another 15 min at 4°C, and then washed twice with PBS. Subsequently, DiR-labelled exosomes were injected into mouse tumors. The DiR-exosomes images were obtained by IVIS Spectrum (Perkin Elmer, USA).

**Haematoxylin and eosin (H&E) and immunohistochemical (IHC) staining**

H&E staining was utilized to observe representative areas, and IHC was performed to observe the expression of the proliferation markers Ki67 (# ab15580, Abcam, USA) and BRD4 (# ab128874, Abcam, USA).

**Supplementary Tables**

**Table S1.** Demographic and clinical characteristics of the subjects who participated in RNA-Seq and proteogenomic analysis

| **Variables** | **RNA-Seq (%), n = 52** | **Proteogenomic (%), n = 25** |
| --- | --- | --- |
| **Age (mean ± SD)** | 67.56 ± 11.04 | 67.60 ± 12.36 |
| **Sex** |  |  |
| Male | 30 (57.7) | 15 (60.0) |
| Female | 22 (42.3) | 10 (40.0) |
| **Smoking status** |  |  |
| Never | 38 (73.1) | 19 (76.0) |
| Ever | 7 (13.5) | 2 (8.0) |
| Current | 4 (7.7) | 3 (12.0) |
| Missing | 3 (5.7) | 1 (4.0) |
| **Drinking status** |  |  |
| Never | 41 (78.8) | 22 (88.0) |
| Ever | 2 (3.8) | 1 (4.0) |
| Current | 6 (11.5) | 2 (8.0) |
| Missing | 3 (5.9) | 0 (0.0) |
| **Tumor Site** |  |  |
| Colon | 30 (57.7) | 15 (60.0) |
| Rectum | 22 (42.3) | 10 (40.0) |
| **Tumor grade** |  |  |
| Well | 2 (3.8) | 0 (0.0) |
| Moderate | 35 (67.3) | 20 (80.0) |
| Poor | 14 (26.9) | 5 (20.0) |
| Missing | 1 (2.0) | 0 (0.0) |
| **Dukes stage** |  |  |
| A | 1 (1.9) | 0 (0.0) |
| B | 26 (50.0) | 12 (48.0) |
| C | 23 (44.2) | 11 (44.0) |
| D | 2 (3.9) | 2 (8.0) |

**Table S2.** Five differentially expressed circRNAs and significant fold changes

| **circRNA Name^a^** | **Chromosome** | **Start site** | **End site** | **Homogenous mRNA** | **Spliced Length** | **\|FC\|** | ***P*** |
| --- | --- | --- | --- | --- | --- | --- | --- |
| hsa_circ_0007379 | chr14 | 35020919 | 35024118 | NONE | 3,199 | 6.27 | 1.41×10^-9^ |
| hsa_circ_0087960 | chr9 | 113734352 | 113735838 | *LPAR1* | 226 | 5.61 | 8.77×10^-8^ |
| hsa_circ_0077837 | chr6 | 131247744 | 131277639 | *EPB41L2* | 824 | 5.31 | 2.35×10^-7^ |
| hsa_circ_0019223 | chr10 | 95790439 | 95792009 | *PLCE1* | 1,570 | 3.46 | 8.23×10^-4^ |
| hsa_circ_0109301 | chr19 | 22157530 | 22171711 | *ZNF208* | 302 | 3.57 | 1.06×10^-3^ |

**^a^** The location information of circRNAs is annotated by the circBase database.

**Table S3.** Demographic and clinical characteristics of the subjects in FISH analysis

| **ID** | **Age** | **Sex** | **Tumor site** | **TNM stage** | **Status** | **Survival time (Months)** |
| --- | --- | --- | --- | --- | --- | --- |
| 1 | 80 | Female | Colon | III | Death | 26 |
| 2 | 61 | Female | Colon | IIIC | Death | 26 |
| 3 | 35 | Male | Colon | IV | Death | 17 |
| 4 | 40 | Male | Colon | IIA | Living | 130 |
| 5 | 81 | Male | Rectum | I | Living | 130 |
| 6 | 62 | Female | Colon | II | Living | 129 |
| 7 | 73 | Female | Rectum | Ⅳ | Death | 27 |
| 8 | 55 | Female | Rectum | III | Death | 74 |
| 9 | 51 | Male | Colon | IIA | Living | 122 |
| 10 | 74 | Female | Rectum | Ⅳ | Death | 35 |
| 11 | 53 | Female | Rectum | I | Living | 121 |
| 12 | 45 | Female | Colon | III | Death | 45 |
| 13 | 65 | Female | Colon | IIA | Living | 117 |
| 14 | 70 | Female | Colon | III | Death | 54 |
| 15 | 51 | Female | Rectum | III | Death | 28 |
| 16 | 73 | Male | Colon | III | Death | 35 |
| 17 | 50 | Female | Colon | II | Death | 15 |
| 18 | 75 | Male | Rectum | II | Death | 51 |
| 19 | 78 | Male | Colon | IV | Death | 55 |
| 20 | 65 | Male | Rectum | Ⅳ | Death | 27 |
| 21 | 76 | Male | Rectum | I | Living | 108 |
| 22 | 52 | Male | Colon | III | Death | 21 |
| 23 | 46 | Male | Colon | IIA | Living | 107 |
| 24 | 56 | Female | Rectum | I | Death | 37 |
| 25 | 63 | Male | Rectum | III | Living | 105 |
| 26 | 64 | Female | Colon | IIIC | Death | 14 |
| 27 | 65 | Female | Rectum | II | Death | 30 |
| 28 | 53 | Male | Colon | IIA | Living | 105 |
| 29 | 79 | Female | Colon | II | Death | 88 |
| 30 | 74 | Male | Rectum | III | Death | 28 |
| 31 | 38 | Female | Rectum | III | Death | 28 |
| 32 | 65 | Male | Rectum | I | Living | 102 |
| 33 | 37 | Male | Colon | II | Living | 102 |
| 34 | 60 | Male | Colon | III | Living | 102 |
| 35 | 78 | Female | Rectum | II | Living | 101 |
| 36 | 64 | Female | Colon | IIA | Living | 101 |
| 37 | 73 | Male | Colon | III | Death | 43 |
| 38 | 64 | Male | Colon | I | Living | 99 |
| 39 | 54 | Female | Colon | II | Living | 98 |
| 40 | 65 | Male | Rectum | I | Living | 98 |
| 41 | 57 | Female | Colon | III | Death | 12 |
| 42 | 62 | Female | Rectum | III | Living | 97 |
| 43 | 33 | Female | Rectum | III | Death | 22 |
| 44 | 61 | Male | Colon | IIA | Living | 97 |
| 45 | 47 | Male | Rectum | I | Living | 97 |
| 46 | 67 | Male | Rectum | I | Living | 94 |
| 47 | 47 | Female | Rectum | Ⅳ | Death | 32 |
| 48 | 54 | Male | Rectum | I | Living | 94 |
| 49 | 68 | Male | Colon | III | Living | 93 |
| 50 | 53 | Male | Rectum | I | Living | 93 |
| 51 | 76 | Male | Colon | IV | Death | 38 |
| 52 | 61 | Male | Rectum | I | Living | 92 |
| 53 | 67 | Female | Rectum | I | Living | 87 |
| 54 | 68 | Male | Rectum | III | Death | 13 |
| 55 | 44 | Male | Rectum | III | Death | 29 |
| 56 | 64 | Male | Rectum | III | Death | 46 |
| 57 | 49 | Female | Colon | IIA | Living | 86 |
| 58 | 50 | Female | Colon | IV | Death | 4 |
| 59 | 58 | Female | Rectum | II | Living | 85 |
| 60 | 85 | Male | Colon | IV | Death | 26 |
| 61 | 63 | Female | Rectum | I | Living | 85 |
| 62 | 81 | Female | Colon | IIA | Living | 85 |
| 63 | 70 | Male | Colon | III | Death | 16 |
| 64 | 52 | Male | Colon | IIA | Living | 85 |
| 65 | 63 | Male | Colon | IV | Death | 13 |
| 66 | 45 | Female | Rectum | III | Death | 21 |
| 67 | 41 | Male | Colon | IIA | Living | 84 |
| 68 | 58 | Male | Colon | IIA | Living | 83 |
| 69 | 86 | Female | Colon | II | Living | 83 |
| 70 | 76 | Female | Colon | IIIB | Living | 81 |
| 71 | 67 | Female | Rectum | III | Death | 66 |
| 72 | 76 | Female | Rectum | III | Death | 27 |
| 73 | 52 | Male | Colon | IIA | Living | 79 |
| 74 | 58 | Male | Colon | IIA | Living | 78 |
| 75 | 53 | Male | Colon | IIA | Death | 68 |
| 76 | 74 | Female | Rectum | I | Living | 78 |
| 77 | 41 | Male | Colon | II | Living | 78 |
| 78 | 62 | Female | Colon | IV | Death | 22 |
| 79 | 48 | Male | Colon | IIA | Living | 77 |

**Table S4.** Demographic and clinical characteristics of subjects with colorectal cancer and cancer-free controls

| **Variables** | **Colorectal cancer (%), n = 112** | **Controls (%), n = 60** |
| --- | --- | --- |
| **Age (mean ± SD)** | 63.72 ± 13.09 | 59.83 ± 11.74 |
| **Sex** |  |  |
| Male | 66 (58.9) | 31 (51.7) |
| Female | 46 (41.1) | 29 (48.3) |
| **Tumor Site** |  |  |
| Colon | 55 (49.1) | - |
| Rectum | 56 (50.0) | - |
| Missing | 1 (0.9) | - |
| **Tumor grade** |  |  |
| Well | 3 (2.7) | - |
| Moderate | 74 (66.1) | - |
| Poor | 29 (25.9) | - |
| Missing | 6 (5.3) |  |
| **Dukes stage** |  |  |
| A | 12 (10.7) | - |
| B | 45 (40.2) | - |
| C | 37 (33.0) | - |
| D | 4 (3.6) | - |
| Missing | 14 (12.5) | - |

**Table S5.** Characteristics of patients with other types of cancer

| **Sample_ID** | **Subject^1^** | **Sex** | **Age** | **Smoking status** | **Drinking status** |
| --- | --- | --- | --- | --- | --- |
| polyps1 | polyps | Male | 64 | NA | NA |
| polyps2 | polyps | Female | 73 | NA | NA |
| polyps3 | polyps | Female | 54 | NA | NA |
| polyps4 | polyps | Female | 62 | NA | NA |
| polyps5 | polyps | Male | 39 | NA | NA |
| polyps6 | polyps | Female | 42 | NA | NA |
| polyps7 | polyps | Female | 64 | NA | NA |
| polyps8 | polyps | Female | 65 | NA | NA |
| polyps9 | polyps | Male | 51 | NA | NA |
| polyps10 | polyps | Male | 55 | NA | NA |
| polyps11 | polyps | Female | 42 | NA | NA |
| polyps12 | polyps | Female | 56 | NA | NA |
| polyps13 | polyps | Male | 64 | NA | NA |
| polyps14 | polyps | Female | 67 | NA | NA |
| polyps15 | polyps | Male | 64 | NA | NA |
| polyps16 | polyps | Female | 51 | NA | NA |
| polyps17 | polyps | Female | 56 | NA | NA |
| polyps18 | polyps | Female | 64 | NA | NA |
| polyps19 | polyps | Female | 46 | NA | NA |
| polyps20 | polyps | Male | 53 | NA | NA |
| polyps21 | polyps | Male | 42 | NA | NA |
| polyps22 | polyps | Male | 51 | NA | NA |
| polyps23 | polyps | Female | 28 | NA | NA |
| polyps24 | polyps | Male | 56 | NA | NA |
| polyps25 | polyps | Female | 70 | NA | NA |
| polyps26 | polyps | Male | 68 | NA | NA |
| polyps27 | polyps | Female | 70 | NA | NA |
| polyps28 | polyps | Male | 68 | NA | NA |
| GC1 | GC | Male | 79 | Ever | Never |
| GC2 | GC | Female | 63 | Never | Never |
| GC3 | GC | Male | 80 | Ever | Never |
| GC4 | GC | Male | 69 | Ever | Never |
| GC5 | GC | Female | 70 | Ever | Never |
| GC6 | GC | Female | 66 | Never | Never |
| GC7 | GC | Female | 65 | Never | Never |
| GC8 | GC | Male | 58 | Never | Never |
| GC9 | GC | Female | 73 | Never | Never |
| GC10 | GC | Female | 73 | Never | Never |
| GC11 | GC | Female | 69 | Ever | Current |
| GC12 | GC | Male | 57 | Ever | Current |
| GC13 | GC | Male | 67 | Never | Never |
| GC14 | GC | Female | 61 | Never | Never |
| GC15 | GC | Male | 55 | Never | Never |
| GC16 | GC | Male | 70 | Never | Never |
| GC17 | GC | Male | 66 | Ever | Never |
| GC18 | GC | Female | 62 | Never | Never |
| GC19 | GC | Male | 56 | Never | Never |
| GC20 | GC | Male | 74 | Current | Never |
| GC21 | GC | Male | 74 | Never | Never |
| GC22 | GC | Male | 56 | Never | Never |
| GC23 | GC | Female | 78 | Never | Never |
| GC24 | GC | Male | 60 | Ever | Never |
| GC25 | GC | Male | 52 | Never | Never |
| GC26 | GC | Male | 69 | Never | Never |
| GC27 | GC | Male | 66 | Never | Never |
| GC28 | GC | Male | 64 | Current | Never |
| GC29 | GC | Male | NA | Ever | Never |
| GC30 | GC | Female | 62 | Never | Never |
| GC31 | GC | Female | 73 | Never | Never |
| GC32 | GC | Female | 55 | Never | Never |
| GC33 | GC | Male | 65 | Never | Never |
| GC34 | GC | Male | 72 | Ever | Never |
| GC35 | GC | Male | 68 | Never | Never |
| GC36 | GC | Male | 60 | Never | Never |
| GC37 | GC | Male | 60 | Never | Never |
| GC38 | GC | Male | 63 | Never | Never |
| GC39 | GC | Female | 60 | Never | Never |
| GC40 | GC | Male | 70 | Never | Never |
| GC41 | GC | Male | 55 | Ever | Never |
| GC42 | GC | Female | 79 | Never | Never |
| GC43 | GC | Male | 73 | Ever | Never |
| GC44 | GC | Male | 75 | Current | Current |
| GC45 | GC | Male | 66 | Never | Never |
| GC46 | GC | Female | 83 | Never | Never |
| GC47 | GC | Male | 78 | Never | Never |
| GC48 | GC | Male | 70 | Ever | Never |
| GC49 | GC | Male | 60 | Ever | Never |
| GC50 | GC | Male | 69 | Never | Never |
| GC51 | GC | Male | 60 | Never | Never |
| GC52 | GC | Male | 68 | Never | Never |
| GC53 | GC | Male | 63 | Never | Never |
| GC54 | GC | Male | 67 | Never | Never |
| GC55 | GC | Male | 76 | Never | Never |
| GC56 | GC | Male | 60 | Never | Never |
| GC57 | GC | Male | 69 | Never | Never |
| GC58 | GC | Female | 66 | Never | Never |
| GC59 | GC | Male | 72 | Ever | Never |
| GC60 | GC | Female | 64 | Never | Never |
| GC61 | GC | Male | 51 | Ever | Never |
| GC62 | GC | Male | 63 | Never | Never |
| GC63 | GC | Male | 72 | Never | Never |
| GC64 | GC | Male | 42 | Ever | Ever |
| GC65 | GC | Female | 60 | Never | Never |
| GC66 | GC | Male | 75 | Current | Ever |
| GC67 | GC | Male | 78 | Never | Never |
| GC68 | GC | Male | 52 | Ever | Ever |
| GC69 | GC | Male | 78 | Never | Never |
| GC70 | GC | Male | 58 | Never | Never |
| GC71 | GC | Male | 59 | Ever | Never |
| GC72 | GC | Male | 64 | Current | Current |
| GC73 | GC | Female | 76 | Never | Never |
| GC74 | GC | Male | 62 | Never | Never |
| BRCA1 | BRCA | Female | 57 | Never | Never |
| BRCA2 | BRCA | Female | 50 | Never | Never |
| BRCA3 | BRCA | Female | 56 | Never | Never |
| BRCA4 | BRCA | Female | 49 | Never | Never |
| BRCA5 | BRCA | Female | 56 | Never | Never |
| BRCA6 | BRCA | Female | 56 | Never | Never |
| BRCA7 | BRCA | Female | 43 | Never | NA |
| BRCA8 | BRCA | Female | 48 | Never | Never |
| BRCA9 | BRCA | Female | 46 | Never | Never |
| BRCA10 | BRCA | Female | 52 | Never | Never |
| BRCA11 | BRCA | Female | 48 | Never | Never |
| BRCA12 | BRCA | Female | 54 | Never | Never |
| BRCA13 | BRCA | Female | 38 | Never | Never |
| BRCA14 | BRCA | Female | 60 | Never | Never |
| BRCA15 | BRCA | Female | 58 | Never | Never |
| BRCA16 | BRCA | Female | 50 | Never | Never |
| BRCA17 | BRCA | Female | 44 | Never | Never |
| BRCA18 | BRCA | Female | 61 | Never | Never |
| BLCA1 | BLCA | Male | 67 | Current | Ever |
| BLCA2 | BLCA | Male | 75 | Current | Ever |
| BLCA3 | BLCA | Male | 72 | Current | Ever |
| BLCA4 | BLCA | Male | 60 | Ever | Ever |
| BLCA5 | BLCA | Male | 70 | Current | Current |
| BLCA6 | BLCA | Male | 70 | Ever | Never |
| BLCA7 | BLCA | Male | 82 | Ever | Ever |
| BLCA8 | BLCA | Male | 81 | Ever | Ever |
| BLCA9 | BLCA | Male | 60 | Ever | Ever |
| BLCA10 | BLCA | Male | 65 | Never | Ever |
| BLCA11 | BLCA | Male | 60 | Never | Never |
| BLCA12 | BLCA | Male | 77 | Never | Never |
| BLCA13 | BLCA | Male | 69 | Current | Ever |
| BLCA14 | BLCA | Male | 56 | Never | Never |
| BLCA15 | BLCA | Female | 69 | Never | Never |
| BLCA16 | BLCA | Male | 73 | Ever | Never |
| BLCA17 | BLCA | Male | 66 | Never | Never |
| BLCA18 | BLCA | Male | 63 | Ever | Ever |
| BLCA19 | BLCA | Male | 69 | Never | Never |
| BLCA20 | BLCA | Male | 63 | Ever | Ever |
| BLCA21 | BLCA | Male | 54 | Ever | Ever |
| BLCA22 | BLCA | Male | 58 | Never | Never |
| BLCA23 | BLCA | Male | 58 | Never | Ever |
| BLCA24 | BLCA | Male | 63 | Never | Never |
| CESC1 | CESC | Female | 49 | Never | Never |
| CESC2 | CESC | Female | 46 | Never | Never |
| CESC3 | CESC | Female | 32 | Never | Never |
| CESC4 | CESC | Female | 55 | Never | Never |
| CESC5 | CESC | Female | 55 | Never | Never |
| CESC6 | CESC | Female | 43 | Never | Never |
| CESC7 | CESC | Female | 39 | Never | Never |
| CESC8 | CESC | Female | 53 | Never | Never |
| CESC9 | CESC | Female | 46 | Never | Never |
| CESC10 | CESC | Female | 60 | Never | Never |
| CESC11 | CESC | Female | 62 | Never | Never |
| CESC12 | CESC | Female | 48 | Never | Never |
| CESC13 | CESC | Female | 49 | Never | Never |
| CESC14 | CESC | Female | 56 | Never | Never |
| CESC15 | CESC | Female | 38 | Never | Never |
| CESC16 | CESC | Female | 42 | Never | Never |
| CESC17 | CESC | Female | 53 | Never | Never |
| CESC18 | CESC | Female | 46 | Never | Never |
| CESC19 | CESC | Female | 61 | Never | Never |
| CESC20 | CESC | Female | 40 | Never | Never |
| CESC21 | CESC | Female | 45 | Never | Never |
| CESC22 | CESC | Female | 44 | Never | Never |
| CESC23 | CESC | Female | 46 | Never | Never |
| CESC24 | CESC | Female | 57 | Never | Never |
| CESC25 | CESC | Female | 68 | Never | Never |
| CESC26 | CESC | Female | 56 | Never | Never |
| CESC27 | CESC | Female | 71 | Never | Never |
| CESC28 | CESC | Female | 55 | Never | Never |
| CESC29 | CESC | Female | 46 | Never | Never |
| CESC30 | CESC | Female | 74 | Never | Never |
| CESC31 | CESC | Female | 73 | Never | Never |
| CESC32 | CESC | Female | 56 | Never | Never |
| KIRC1 | KIRC | Female | 71 | Never | Never |
| KIRC2 | KIRC | Male | 86 | Never | Never |
| KIRC3 | KIRC | Male | 72 | Never | Never |
| KIRC4 | KIRC | Female | 57 | Never | Never |
| KIRC5 | KIRC | Male | 66 | Never | Never |
| KIRC6 | KIRC | Male | 51 | Never | Ever |
| KIRC7 | KIRC | Male | 50 | Never | Never |
| KIRC8 | KIRC | Male | 65 | Current | Never |
| KIRC9 | KIRC | Male | 62 | Ever | Current |
| KIRC10 | KIRC | Male | 53 | Never | Never |
| KIRC11 | KIRC | Female | 57 | Never | Never |
| KIRC12 | KIRC | Male | 54 | Never | Never |
| KIRC13 | KIRC | Male | 64 | Never | Never |
| KIRC14 | KIRC | Female | 54 | Never | Never |
| KIRC15 | KIRC | Male | 65 | Never | Never |
| KIRC16 | KIRC | Male | 79 | Never | Never |
| KIRC17 | KIRC | Male | 65 | Never | Never |
| KIRC18 | KIRC | Female | 67 | Never | Never |
| KIRC19 | KIRC | Male | 75 | Never | Never |
| LUAD1 | LUAD | Male | 59 | Yes | No |
| LUAD2 | LUAD | Male | 63 | Yes | Yes |
| LUAD3 | LUAD | Male | 67 | No | No |
| LUAD4 | LUAD | Male | 65 | Yes | No |
| LUAD5 | LUAD | Female | 62 | No | No |
| LUAD6 | LUAD | Female | 65 | No | No |
| LUAD7 | LUAD | Male | 54 | Yes | Yes |
| LUAD8 | LUAD | Female | 48 | No | No |
| LUAD9 | LUAD | Male | 51 | No | No |
| LUAD10 | LUAD | Male | 58 | Yes | No |
| LUAD11 | LUAD | Female | 40 | No | No |
| LUAD12 | LUAD | Male | 59 | No | No |
| LUAD13 | LUAD | Male | 52 | No | No |
| LUAD14 | LUAD | Male | 63 | Yes | No |
| LUAD15 | LUAD | Male | 64 | No | No |
| LUAD16 | LUAD | Female | 70 | No | No |
| LUAD17 | LUAD | Male | 65 | Yes | Yes |
| LUAD18 | LUAD | Female | 56 | No | No |
| LUAD19 | LUAD | Male | 62 | No | No |
| LUAD20 | LUAD | Male | 64 | Yes | Yes |
| LUAD21 | LUAD | Male | 56 | Yes | Yes |
| LUAD22 | LUAD | Male | 58 | Yes | No |
| LUAD23 | LUAD | Male | 67 | No | No |
| LUAD24 | LUAD | Male | 77 | Yes | Yes |
| LUAD25 | LUAD | Male | 62 | Yes | Yes |
| LUAD26 | LUAD | Male | 37 | Yes | No |
| LUAD27 | LUAD | Male | 68 | Yes | No |
| LUAD28 | LUAD | Female | 66 | No | No |
| LUAD29 | LUAD | Male | 60 | No | Yes |
| LUAD30 | LUAD | Male | 69 | Yes | No |
| LUAD31 | LUAD | Male | 52 | Yes | No |
| LUAD32 | LUAD | Female | 72 | No | No |
| LUAD33 | LUAD | Male | 68 | Yes | No |
| LUAD34 | LUAD | Male | 57 | Yes | No |
| LUAD35 | LUAD | Female | 49 | No | No |
| LUAD36 | LUAD | Male | 73 | No | No |
| LUAD37 | LUAD | Female | 67 | No | No |
| LUAD38 | LUAD | Male | 65 | Yes | Yes |
| LUAD39 | LUAD | Male | 57 | Yes | Yes |
| LUAD40 | LUAD | Male | 71 | Yes | No |
| LUAD41 | LUAD | Female | 67 | No | No |
| LUAD42 | LUAD | Female | 64 | No | No |

^1^GC, gastric carcinoma; BRCA, breast invasive carcinoma; BLCA, bladder urothelial carcinoma；CESC, cervical squamous cell carcinoma and endocervical adenocarcinoma; KIRC, kidney renal clear cell carcinoma; LUAD, lung adenocarcinoma.

NA means missing.

**Table S6.** Demographic and clinical characteristics of the colorectal cancer patients before and after operation

| **ID** | **Age** | **Sex** | **Tumor site** | **Tumor grade** | **Dukes stage** | **Smoking status** |
| --- | --- | --- | --- | --- | --- | --- |
| 1 | 29 | Female | Colon | Poor | B | Never |
| 2 | 83 | Female | Colon | Moderate | B | Never |
| 3 | 23 | Female | Colon | Poor | B | Never |
| 4 | 85 | Female | Colon | Poor | B | Never |
| 5 | 53 | Male | Colon | Poor | B | Current |
| 6 | 77 | Male | Colon | Poor | B | NA |
| 7 | 29 | Female | Rectum | Moderate | C | Never |
| 8 | 44 | Female | Colon | Moderate | C | Never |
| 9 | 21 | Male | Colon | Moderate | C | Never |
| 10 | 61 | Male | Colon | Moderate | C | Ever |
| 11 | 60 | Female | Colon | Poor | C | Never |
| 12 | 85 | Female | Colon | Moderate | C | Never |
| 13 | 82 | Female | Colon | Moderate | C | Never |
| 14 | 72 | Female | Colon | Moderate | B | Never |
| 15 | 64 | Male | Rectum | Moderate | C | Never |
| 16 | 66 | Female | Colon | Moderate | A | Never |
| 17 | 61 | Male | Colon | Moderate | B | Never |
| 18 | 77 | Female | Colon | Poor | B | Never |
| 19 | 68 | Male | Rectum | Poor | C | Never |
| 20 | 85 | Male | Colon | Moderate | C | Never |
| 21 | 74 | Male | Rectum | Moderate | B | Never |
| 22 | 29 | Male | Colon | Moderate | B | Current |
| 23 | 53 | Female | Colon | Moderate | B | Never |
| 24 | 71 | Male | Rectum | Moderate | C | Never |
| 25 | 38 | Female | Colon | Moderate | C | Never |

NA means missing.

**Table S7.** Characterization of circLPAR1-binding proteins

| **No** | **Protein Name** | **Protein Score** | **Protein Mass** | **Protein Peptide** | **Protein Coverage** |
| --- | --- | --- | --- | --- | --- |
| 1 | Major Vault Protein (MVP) | 516 | 99551 | 12 | 16.1 |
| 2 | RNA-binding protein FUS (FUS) | 221 | 53622 | 3 | 8.9 |
| 3 | Myosin-9 (MYH9) | 153 | 227646 | 1 | 0.9 |
| 4 | Zinc finger and BTB domain-containing protein 7A (ZBT7A) | 132 | 62199 | 2 | 7.2 |
| 5 | Splicing factor 3A subunit 3 (SF3A3) | 118 | 59154 | 1 | 4.2 |
| 6 | Eukaryotic translation initiation factor 3 subunit h (eIF3h) | 73 | 40076 | 1 | 3.4 |
| 7 | Actin, cytoplasmic 1 (ACTB) | 71 | 42052 | 1 | 4.8 |
| 8 | 60S ribosomal protein L8 (RL8) | 56 | 28235 | 1 | 4.3 |
| 9 | Eukaryotic translation initiation factor 3 subunit f (eIF3f) | 54 | 37654 | 1 | 4.8 |
| 10 | Erlin-1 (ERLN1) | 53 | 39318 | 1 | 3.4 |
| 11 | Splicing factor 3B subunit 3 (SF3B3) | 50 | 136575 | 1 | 1.2 |
| 12 | Peroxiredoxin-4 (PRDX4) | 44 | 30749 | 1 | 4.4 |
| 13 | Nucleolar protein 6 (NOL6) | 44 | 128368 | 1 | 1.4 |
| 14 | Eukaryotic translation initiation factor 4E type 2 (IF4E2) | 36 | 28458 | 1 | 6.5 |
| 15 | Eukaryotic translation initiation factor 3 subunit e (eIF3e) | 33 | 52587 | 1 | 2.2 |
| 16 | 60S ribosomal protein L27a (RL27A) | 33 | 16665 | 1 | 7.4 |
| 17 | G patch domain-containing protein 2-like (GPT2L) | 27 | 55138 | 1 | 2.3 |
| 18 | Protein mago nashi homolog 2 (MGN2) | 26 | 17322 | 1 | 13.5 |
| 19 | 60S ribosomal protein L17 (RL17) | 26 | 21611 | 1 | 5.4 |
| 20 | 40S ribosomal protein S3 (RS3) | 26 | 26842 | 1 | 6.6 |
| 21 | Zinc finger protein 518A (Z518A) | 24 | 169071 | 1 | 0.6 |
| 22 | Proto-oncogene serine/threonine-protein kinase mos (MOS) | 22 | 38366 | 1 | 2.6 |
| 23 | Transcription elongation factor SPT4 (SPT4H) | 19 | 13470 | 1 | 6 |

**Table S8.** The correlation between protein and its parental gene expression and prediction of RNA binding ability

| **No** | **Protein name** | **Gene name** | ***r*** | ***P*** | **catRAPID ^signature^** |  | **catRAPID ^express^** | |
| --- | --- | --- | --- | --- | --- | --- | --- | --- |
|  |  |  |  |  | **score** |  | **interaction propensity (IP)** | **discrimination power (DP)** |
| 1 | SF3A3 | *SF3A3* | 0.721 | 3.47×10^-9^ | 0.48 |  | 16 | 45% |
| 2 | eIF3h | *EIF3H* | 0.662 | 1.65×10^-7^ | 0.68 |  | 24 | 64% |
| 3 | MGN2 | *MAGOHB* | 0.538 | 1.03×10^-3^ | 0.47 |  | 18 | 50% |
| 4 | RL27A | *RPL27A* | 0.405 | 3.52×10^-3^ | 0.76 |  | 24 | 63% |
| 5 | SPT4H | *SUPT4H1* | -0.344 | 1.45×10^-2^ | 0.67 |  | 13 | 38% |
| 6 | IF4E2 | *EIF4E2* | -0.281 | 4.82×10^-2^ | 0.76 |  | 5 | 22% |

**Table S9.** Sequences of circLPAR1 used for FISH analysis in the study

| **Gene name** | **Sequences (5'-3')** |
| --- | --- |
| 5-FAM-labeled circLPAR1_probe | TCCACC+TGGGGC+TGTGAAATTACAG |
| 5-FAM-labeled circLPAR1_probe1 | ATCAGACG+TCCACC+TGGGGCTGTGA |
| 5-FAM-labeled circLPAR1_probe2 | ATAAA+TCAGACG+TCCACCTGGGGCT |
| Cy3-labeled circLPAR1_probe | GTCCACC+TGGGGCTG+TGAAATTACA |
| Cy3-labeled circLPAR1_probe1 | AAATCAGACG+TCCACC+TGGGGCTGT |
| Cy3-labeled circLPAR1_probe2 | ATCAGACG+TCCACC+TGGGGCTGTGA |

**Table S10.** Sequences of primers used for RT-qPCR in the study

| **Gene name** | **Primer** | **Sequences (5'-3')** |
| --- | --- | --- |
| hsa_circ_0007379 | Forward | GCAGATGATGAGAAATCACAGAG |
|  | Reverse | TGCCTGTAACAACGATCACA |
| circLPAR1 | Forward | TGTTCACCACCTACAACCAC |
|  | Reverse | GAGAAGCTGTGTACCTGATGC |
| Linear *LPAR1* | Forward | GCCACCTTTAGGCAGATCCT |
|  | Reverse | AGCCAAGATGGTGTGGTTGA |
| hsa_circ_0077837 | Forward | AAACATGCCAAGGGACAAGT |
|  | Reverse | CTTCAGACACAGAGCCTACTTC |
| *BRD4* | Forward | GAGCTACCCACAGAAGAAACC |
|  | Reverse | GAGTCGATGCTTGAGTTGTGTT |
| *U6* | Forward | CTCGCTTCGGCAGCACA |
|  | Reverse | AACGCTTCACGAATTTGCGT |
| *GAPDH* | Forward | GGAGCGAGATCCCTCCAAAAT |
|  | Reverse | GGCTGTTGTCATACTTCTCATGG |
| has-miR-762 | Forward | ACACTCCAGCTGGGGGGGCTGGGGCCGG |
|  | Reverse | TGGTGTCGTGGAGTCG |
|  | RT | CTCAACTGGTGTCGTGGAGTCGGCAATTCAGTTGAGGCTCGGCC |
| U6 | Forward | CGCTTCGGCAGCACATATACTAAAATTGGAAC |
|  | Reverse | GCTTCACGAATTTGCGTGTCATCCTTGC |
|  | RT | AAAATATGGAACGCTTCACG |

**Figure legends**

Fig. S1. The expression of circRNAs detected by RT-qPCR. The expression of hsa_circ_0007379 **(A)** and hsa_circ_0077837 **(B)** in normal colorectal mucosal cells (FHC) and colorectal cancer cells (HCT116 and DLD1) detected by RT-qPCR. (C-D) The expression of circLPAR1 in cellular culture medium after treating with exosome secretion inhibitor GW4869 (circLPAR1-GW4869) or negative control DMSO (circLPAR1-DMSO). Statistical significance was assessed using two-tailed Student’s *t*-test. The values represent the mean ± SD. ^*^*P* < 0.05.


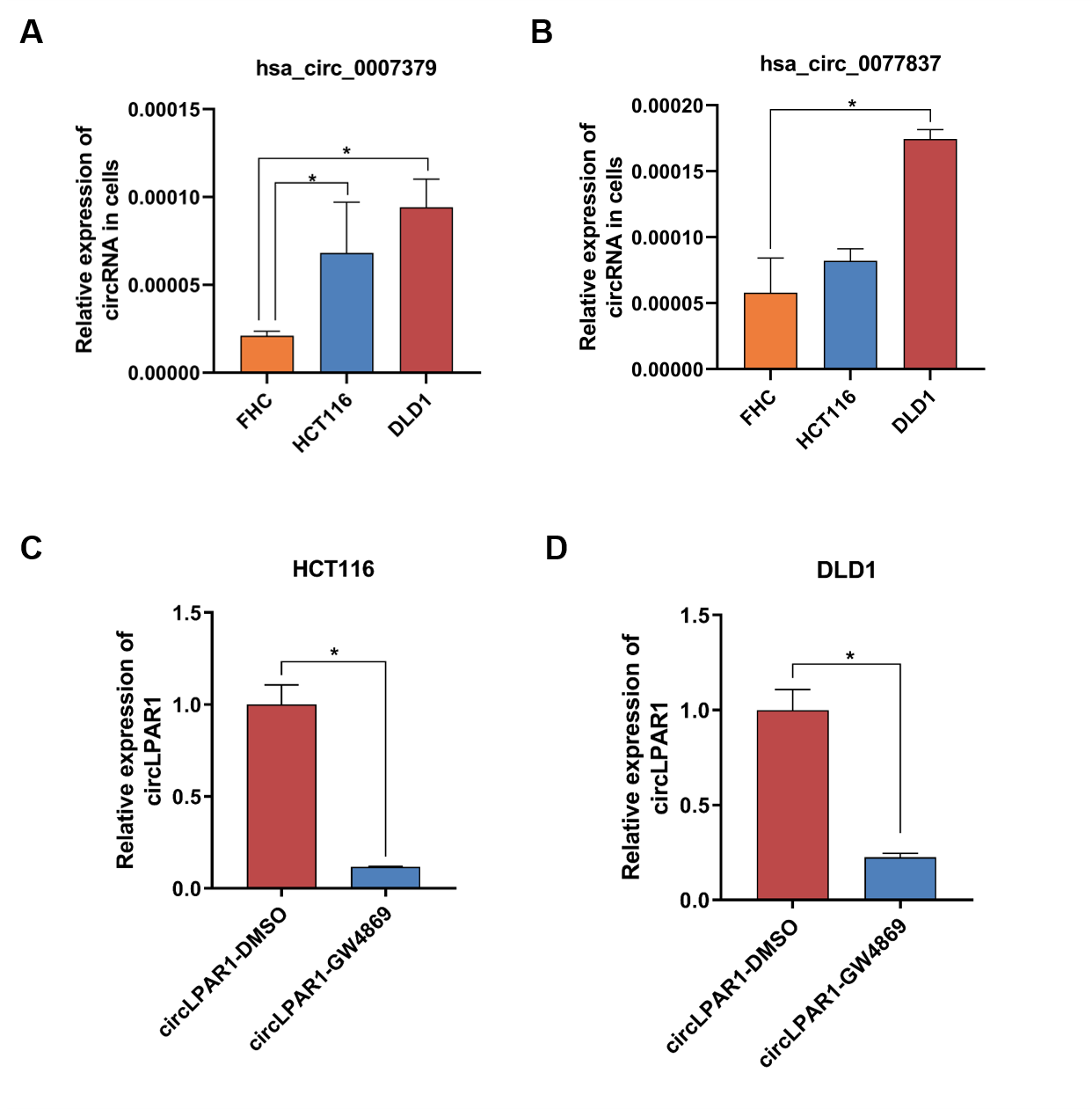


Fig. S2. The expression of circLPAR1 in CRC cells treated with actinomycin D and colorectal tumors. (A) The abundance of circLPAR1 and *LPAR1* mRNA in DLD1 cells treated with actinomycin D at the indicated time points. Statistical significance was assessed using two-tailed Student’s *t*-test. The values represent the mean ± SD. (B) The circLPAR1 levels in colorectal cancer tissue samples were measured by FISH analysis.


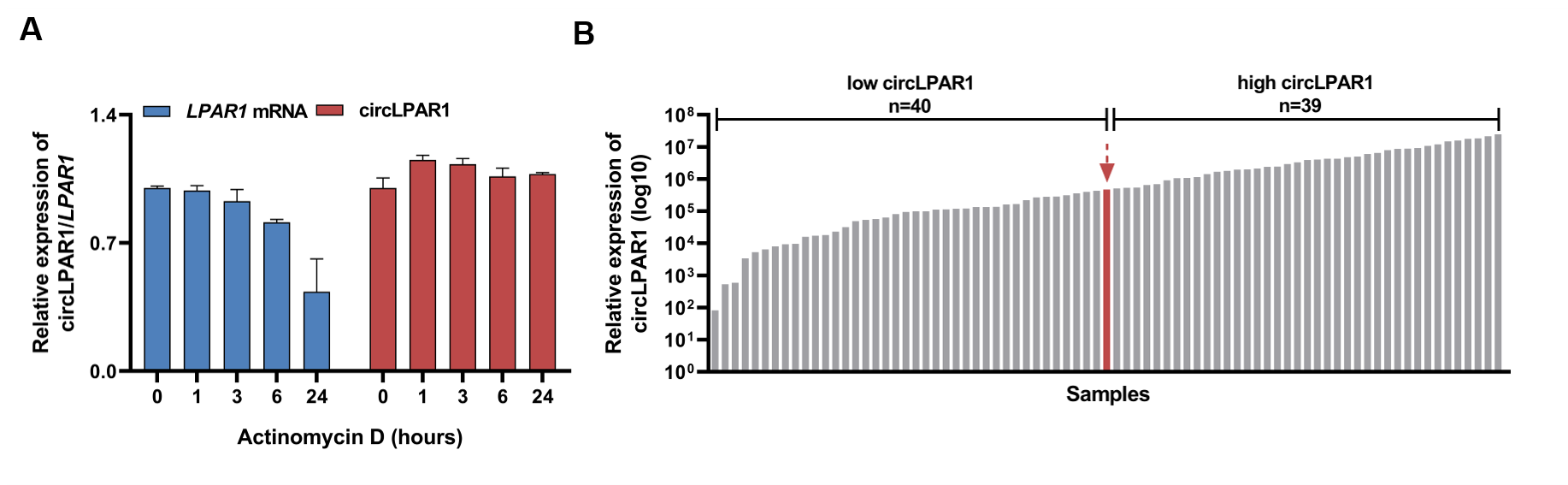


Fig. S3. Characterization of exosomes derived from human plasma. The exosomes isolated from plasma of cancer-free controls, patients with polyps and colorectal cancer are designated control-Exos, polyps-Exos and CRC-Exos, respectively. (A) Visualization of purified control/polyps/CRC-Exos. TEM was used to confirm the shape of exosomes. Scale bar, 100 nm. **(B)** Western blot showing exosome-specific markers TSG101 and Alix. **(C)** The size distributions of control/polyps/CRC-Exos. The exosome was identified by NanoFCM. The X-axis represents exosome size and the Y- axis represents exosome number.


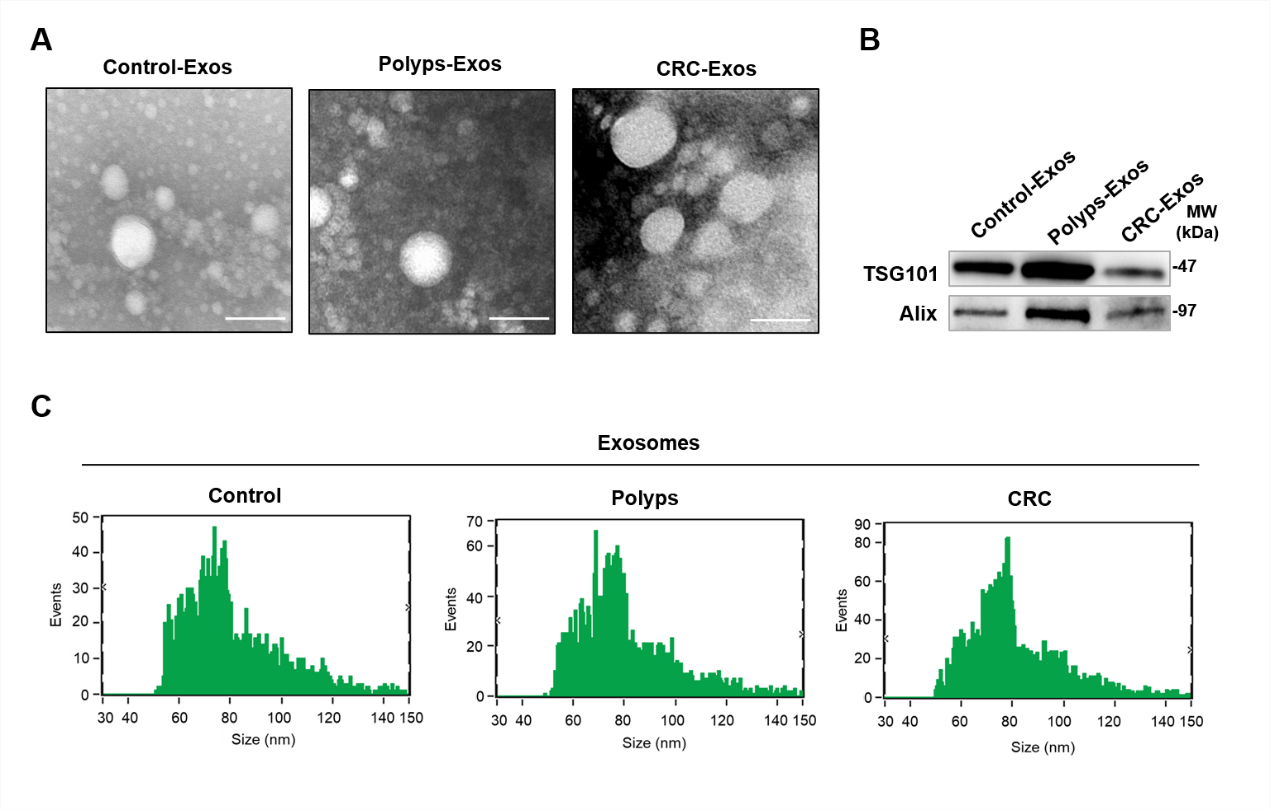


Fig. S4. The expression of traditional clinical biomarkers in plasma. Differential expression of CEA (A) and CA19-9 (B) between colorectal cancer patients and cancer-free controls. Statistical significance was assessed using two-tailed Student’s *t*-test. The values represent the mean ± SD. ^*^*P* < 0.05.


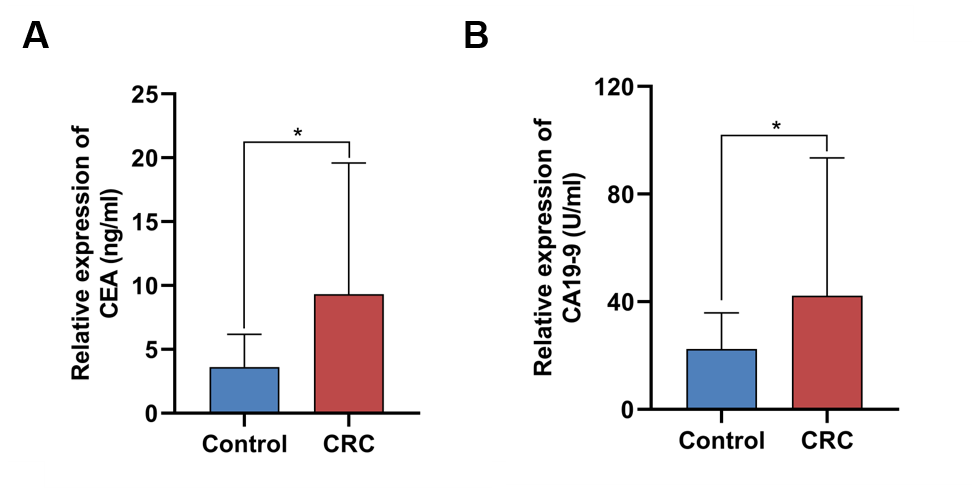


Fig. S5. **ROC curve analysis of exosomal circLPAR1 and** traditional clinical biomarkers **in human plasma.**


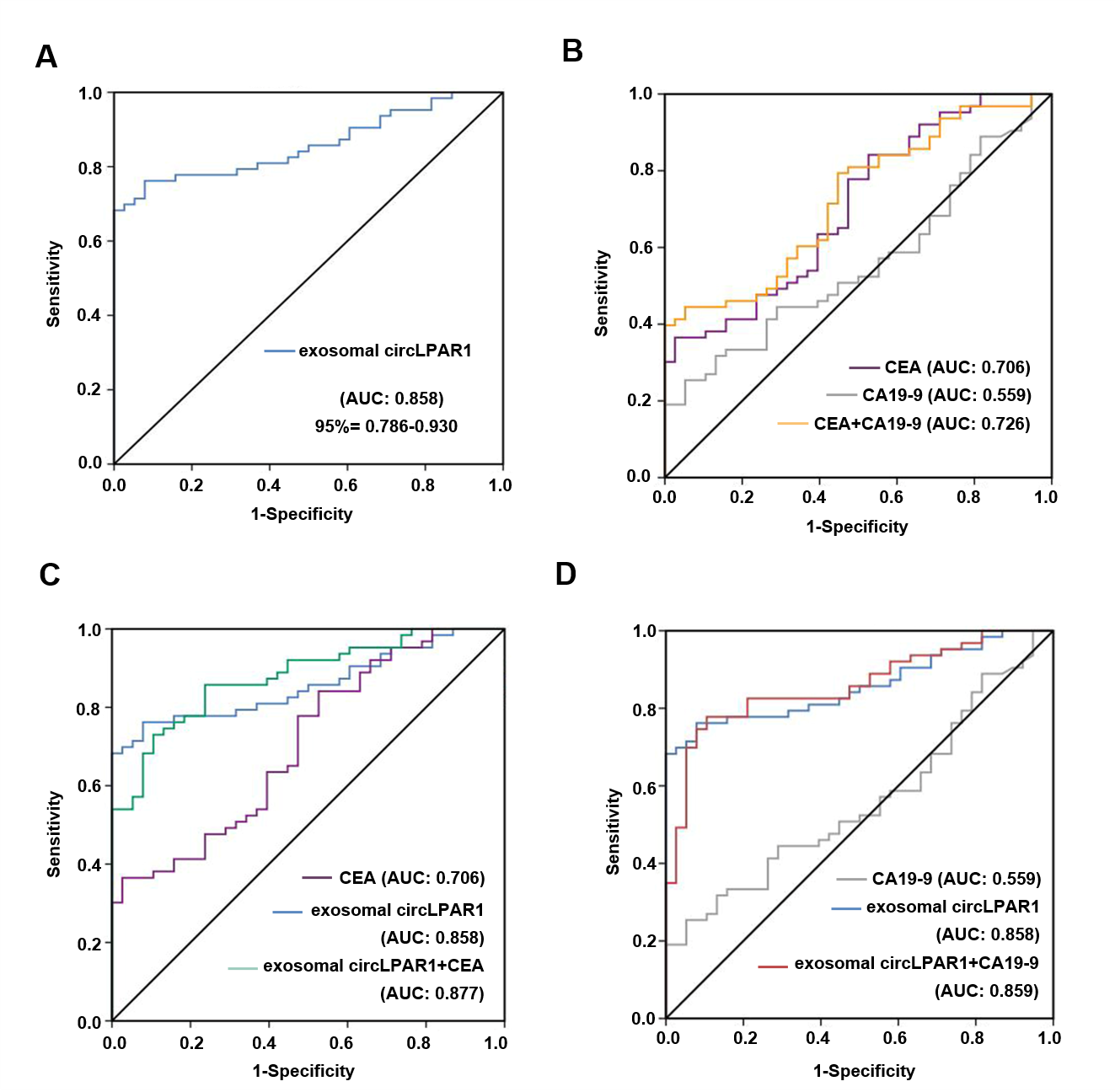


Fig. S6. **Fluorescence observation of colorectal cancer cells after incubation with PKH67-labelled exosomes or non-exosomes.** Representative fluorescence images of colorectal cancer cells after incubation with HCT116/DLD1-Exos labelled with PKH67 (green) or non-Exos. Scale bar, 25 μm.


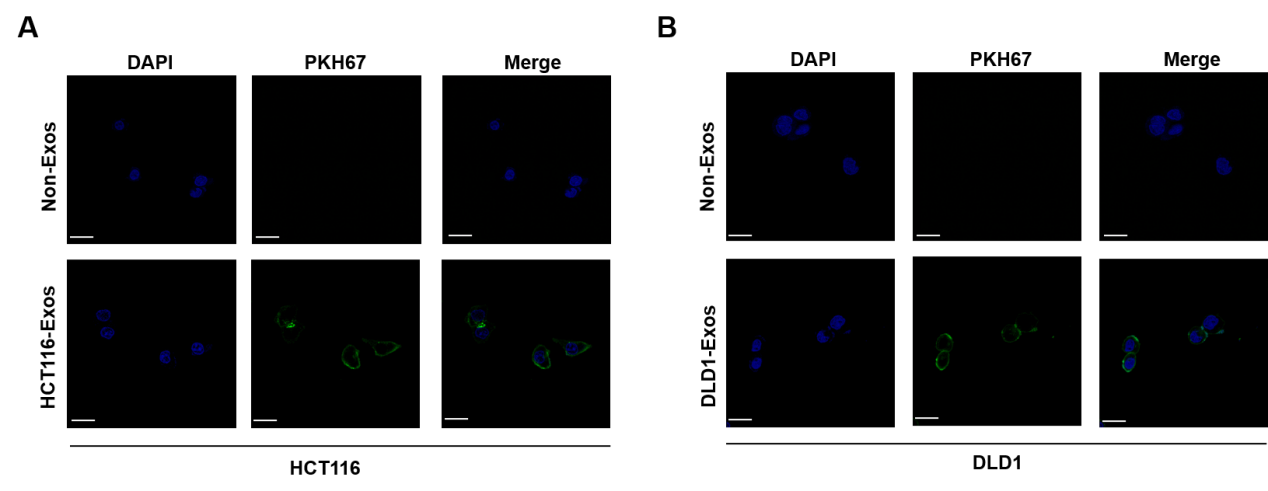


Fig. S7. **Fluorescence observation of colorectal cancer cells incubation with PKH67-labelled exosomes pretreated with cytochalasin D.** Representative images of colorectal cancer cell fluorescence after incubation with PKH67- labelled exosomes pre-treated with cytochalasin D (CytoD). Scale bar, 25 μm.


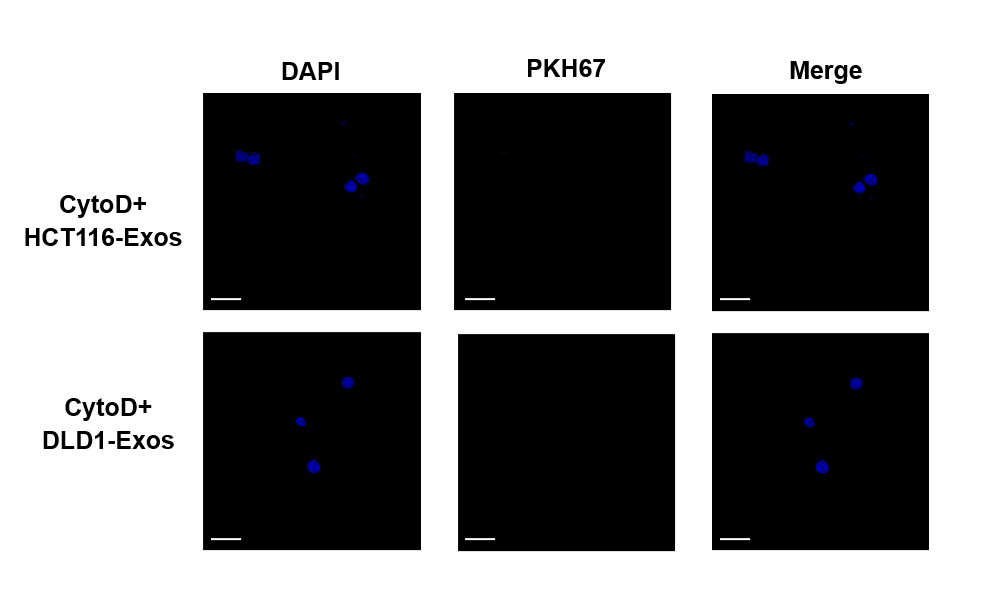


Fig. S8. The expression of circLPAR1 in exosomes derived from colorectal cancer cells. Exosomes were isolated from HCT116 or DLD1 cells transfected with circLPAR1/controls expression plasmid, namely circLPAR1-Exos and NC-Exos, respectively. Differential expression of circLPAR1 between circLPAR1-Exos and NC-Exos. Statistical significance was assessed using two-tailed Student’s *t*-test. The values represent the mean ± SD. ^*^*P* < 0.05.

**
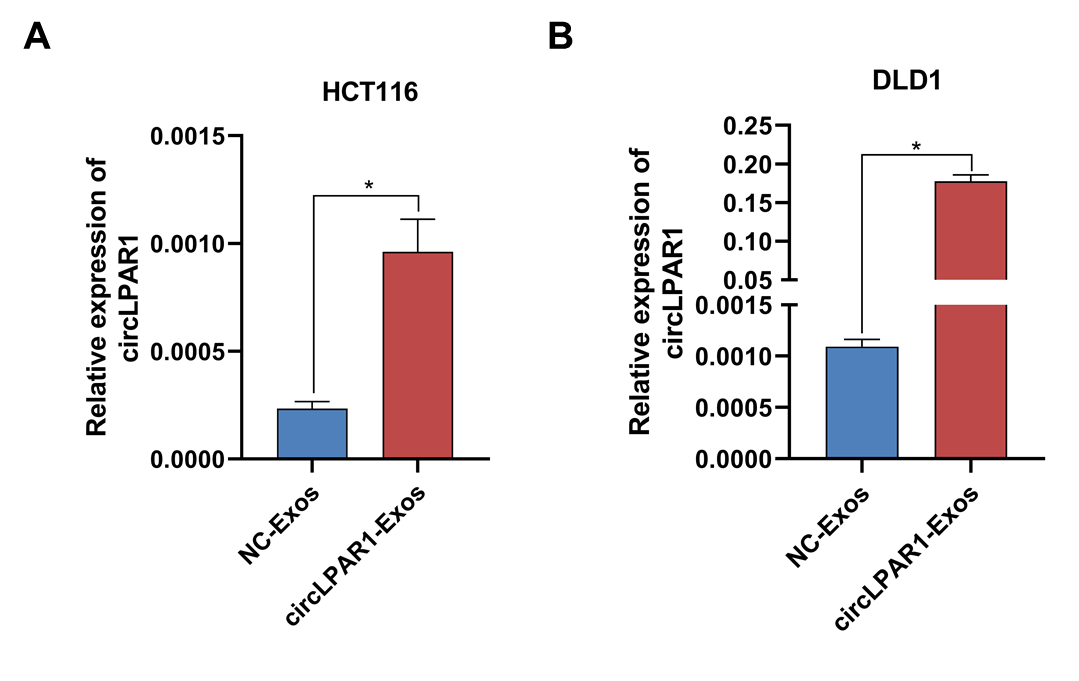
**

Fig. S9. The effect of exosomal circLPAR1 on invasion and migration abilities of HCT116 cells. Exosomes were isolated from HCT116 cells transfected with circLPAR1/control expression plasmid, namely circLPAR1-Exos and NC-Exos, respectively. **(A)** Representative images of invasion and migration assays of HCT116 cells after incubation with circLPAR1/NC-Exos. Scale bar, 100 μm. **(B)** The number of cells was counted. Statistical significance was assessed using two-tailed Student’s *t*-test. The values represent the mean ± SD. ^*^*P* < 0.05.

**
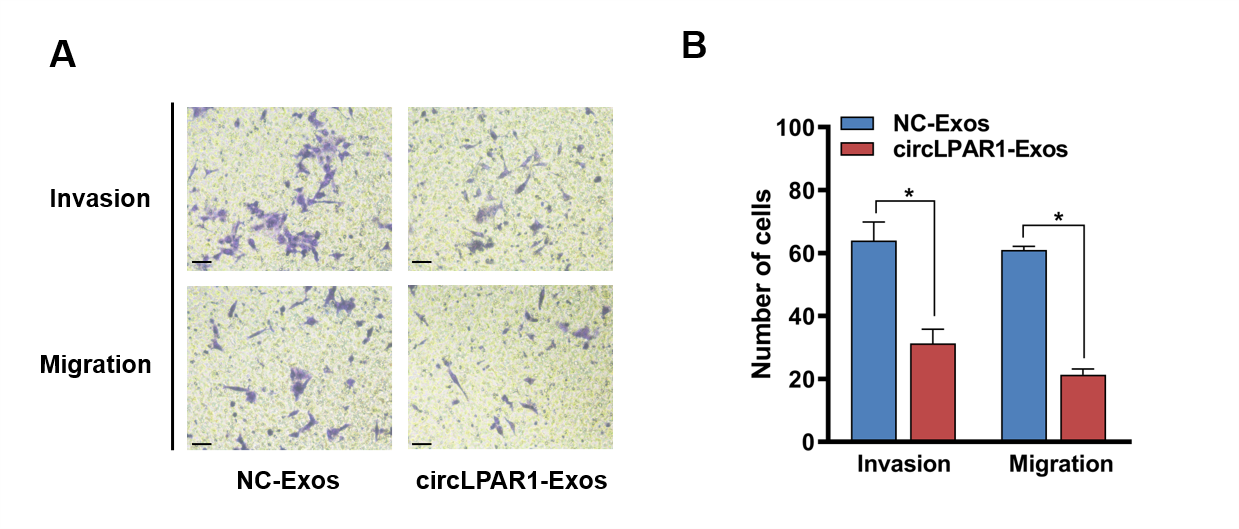
**

Fig. S10. The effect of circLPAR1 overexpression on colorectal cancer cellular phenotypes. The circLPAR1 or NC vector was transfected into HCT116 and DLD1 cells, namely circLPAR1 over and NC, respectively. **(A)** The ectopic expression of circLPAR1 in colorectal cancer cells. (B) The role of circLPAR1 on the proliferation of HCT116 (left) and DLD1 (right) cells as measured using a CCK-8 assay. (C) The role of circLPAR1 on the clone formation ability of HCT116 (left) and DLD1 (right) cells. (D) The role of circLPAR1 on the invasion (left) and migration (right) abilities detected by a transwell assay. Scale bar, 100 μm. Statistical significance was assessed using two-tailed Student’s *t*-test. The values represent the mean ± SD. ^*^*P* < 0.05.


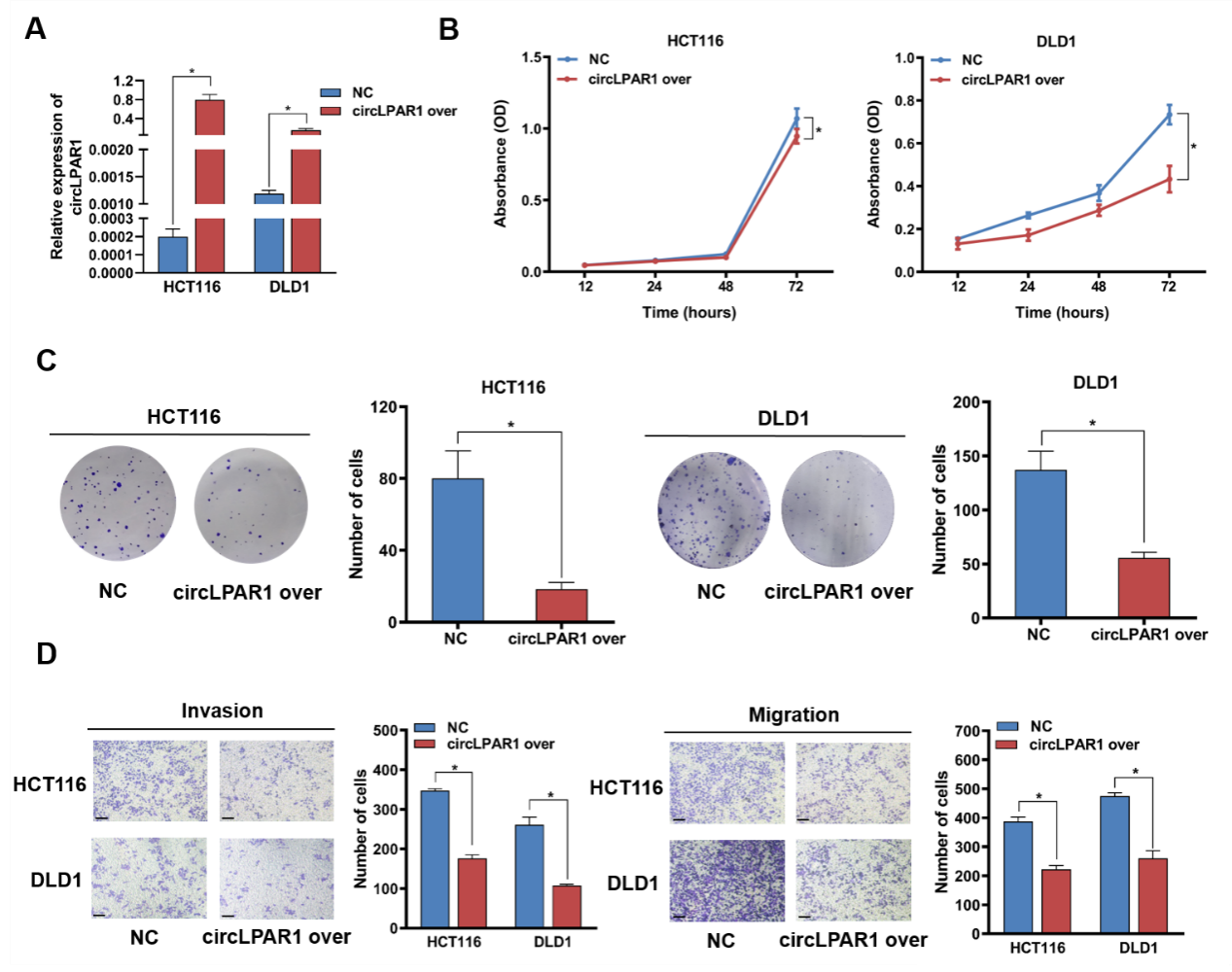


Fig. S11. The cellular localization of circLPAR1 in colorectal cancer cells. 18S was used as a cytoplasmic marker. U6 was used as a nuclear marker. (A) FISH analysis showing the cellular location of 18S, circLPAR1 and U6 in HCT116 (left) and DLD1(right) cells. Scale bar, 25μm. (B) The expression of circLPAR1 in nucleus and cytoplasm of HCT116 (left) or DLD1(right). *GAPDH* served as a positive control for the cytoplasm and *U6* served as a positive control for the nucleus. The values represent the mean ± SD.


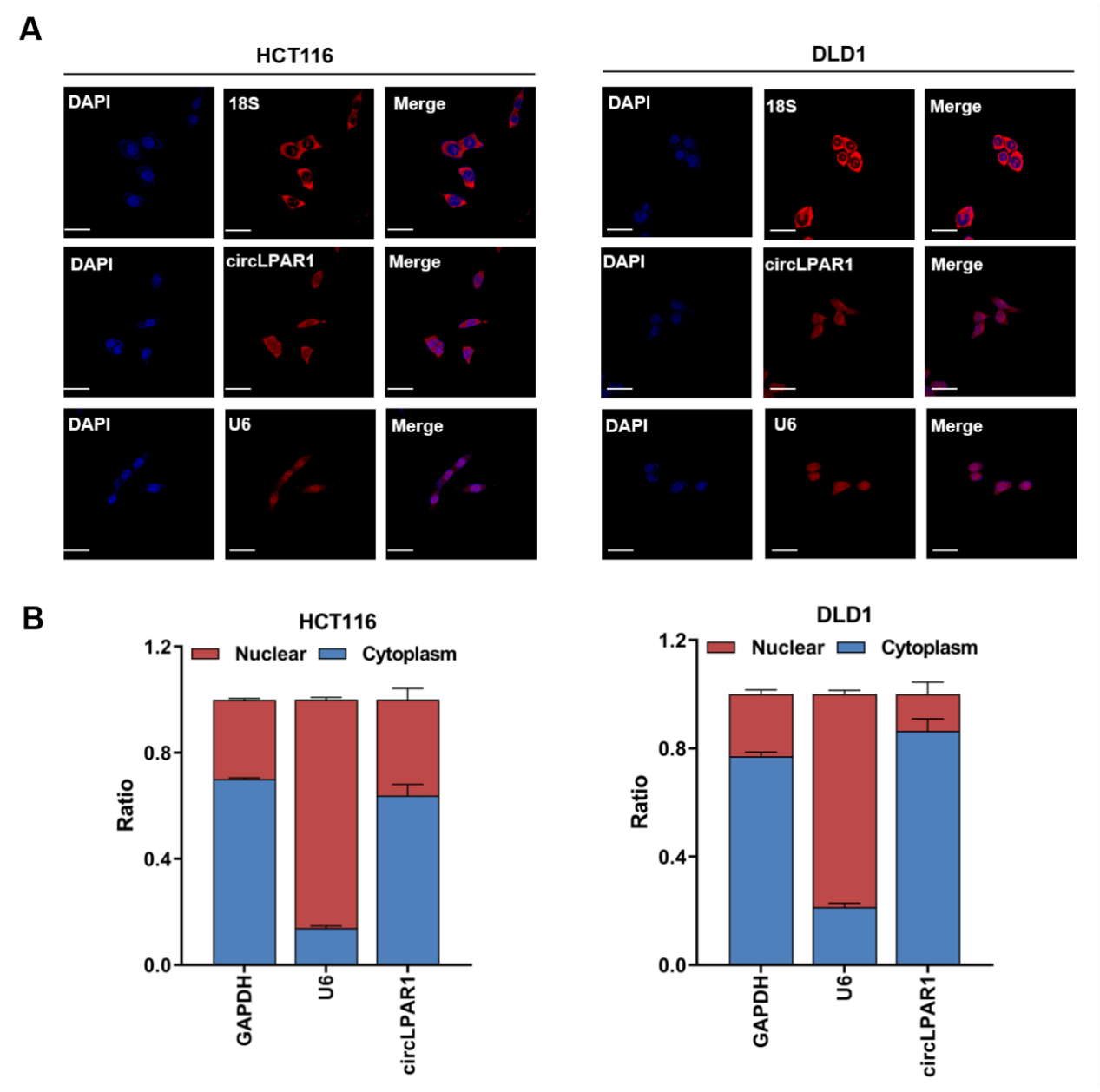


Fig. S12. MS2-CP-Flag was pulled down by anti-flag and evaluated by Western blot.


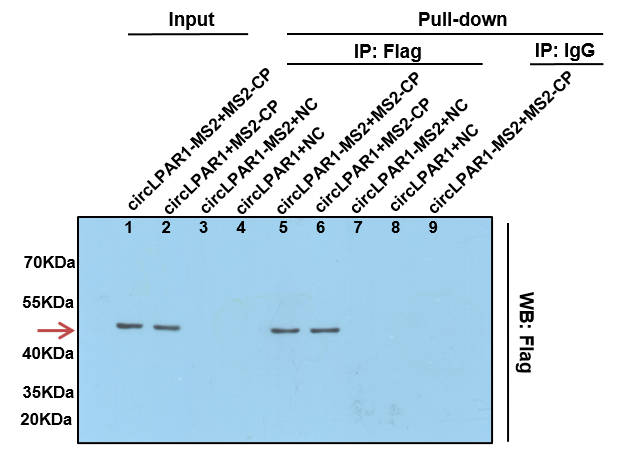


Fig. S13. The **proteomic analysis showing** 23 proteins levels based on 25 paired colorectal cancer tissues. The expression of GPT2L, Z518A and MOS proteins was not observed in proteomic analysis. Statistical significance was assessed using two-tailed Student’s *t*-test. The values represent the mean ± SD. ^*^*P* < 0.05.


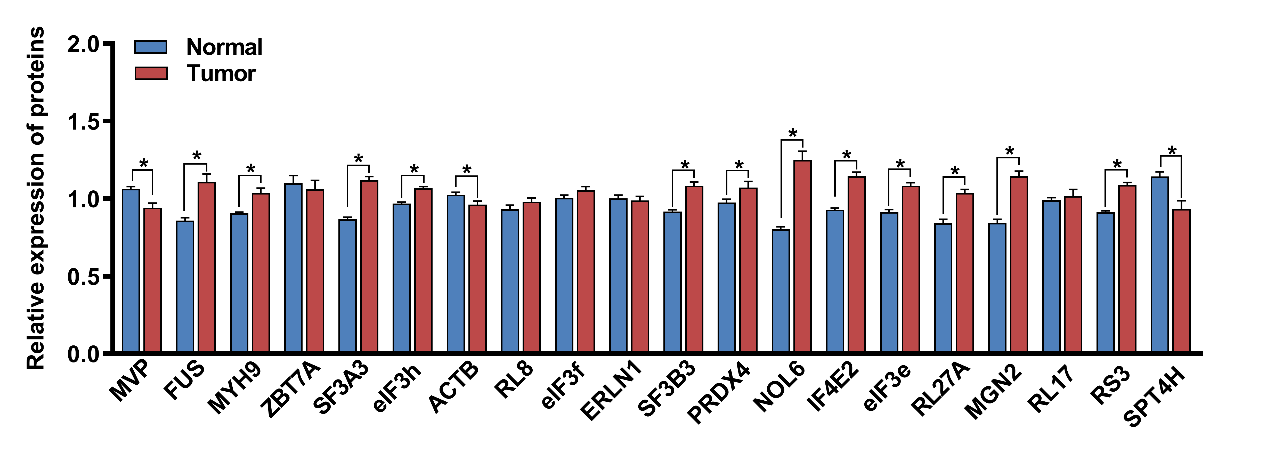


Fig. S14. Mass spectrometry analysis identification of eIF3h.


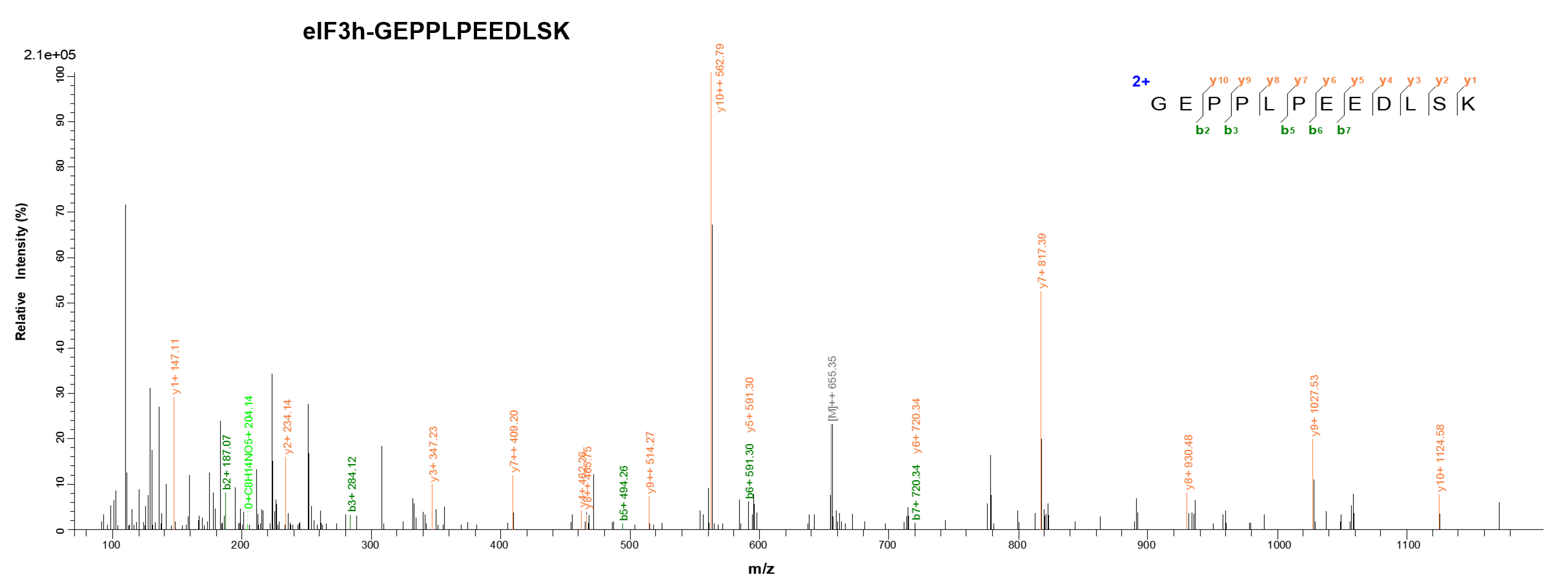


Fig. S15. The effect of *BRD4* regulation by circLPAR1 on colorectal cancer cellular phenotype. The circLPAR1 lentiviral vector was transfected into DLD1 cells, which were designated circLPAR1. The *BRD4*-overexpression plasmid was

transfected into circLPAR1 cells, which were designated circLPAR1+*BRD4*; then, circLPAR1+*BRD4* cells were treated with AZD5153 and designated circLPAR1+*BRD4+*AZD5153. The ectopic expression of *BRD4* at the mRNA levels and protein levels determined by RT-qPCR **(A)** and Western blot **(B)** in circLPAR1 and circLPAR1+*BRD4* cells. (C) Western blot showing BRD4 protein expression levels after treatment with AZD5153. (D) Western blot showing BRD4 protein expression levels in circLPAR1, circLPAR1+*BRD4* and circLPAR1+*BRD4+*AZD5153 cell lines. (E) The proliferation of the indicated cells as measured using a CCK-8 assay. **(F)** The invasion and migration abilities of the indicated cells detected using a transwell assay. Scale bar, 100 μm. Statistical significance was assessed using two-tailed Student’s *t*-test. The values represent the mean ± SD. ^*^*P* < 0.05.


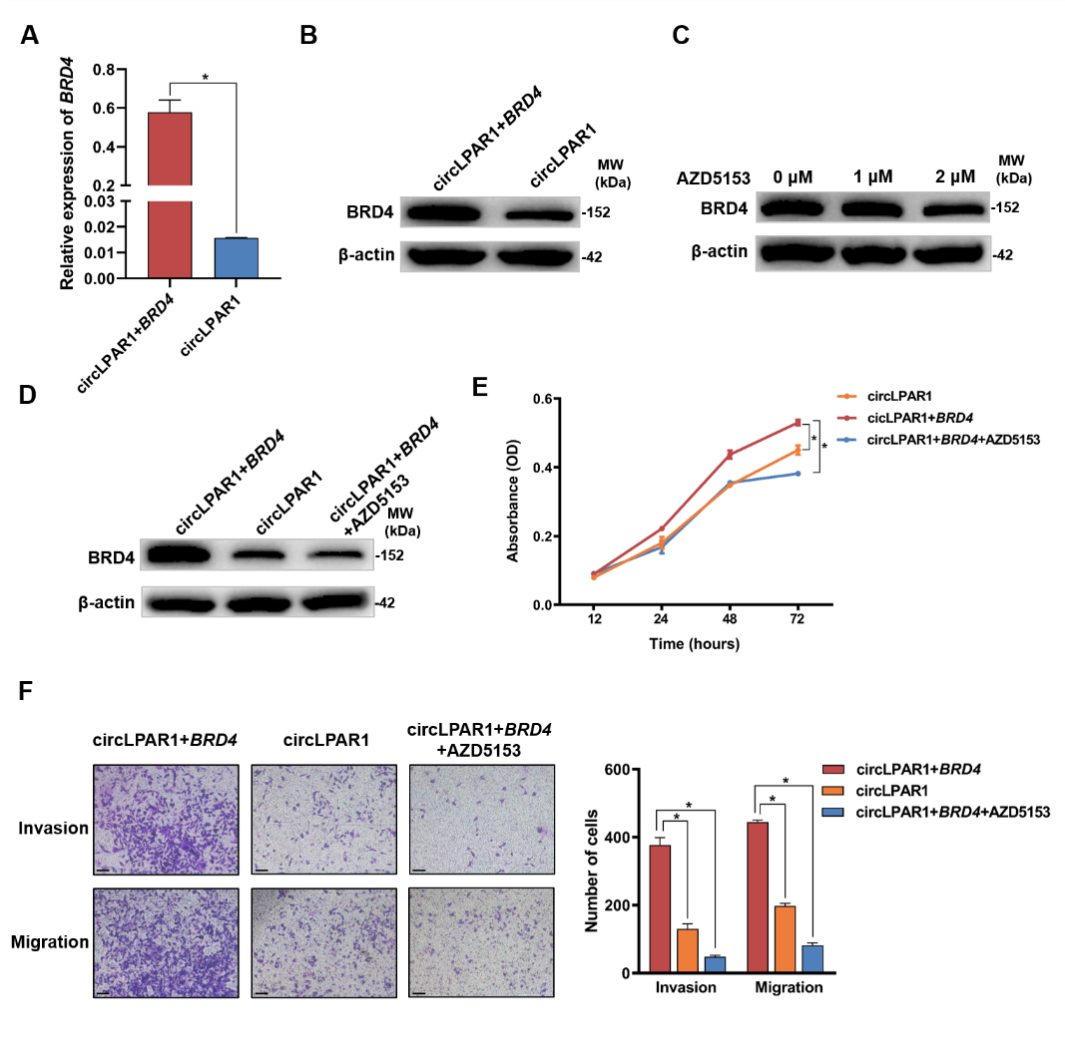


Fig. S16. **The enrichment of** miR-762 **in the complex with MS2-CP-Flag.**

**
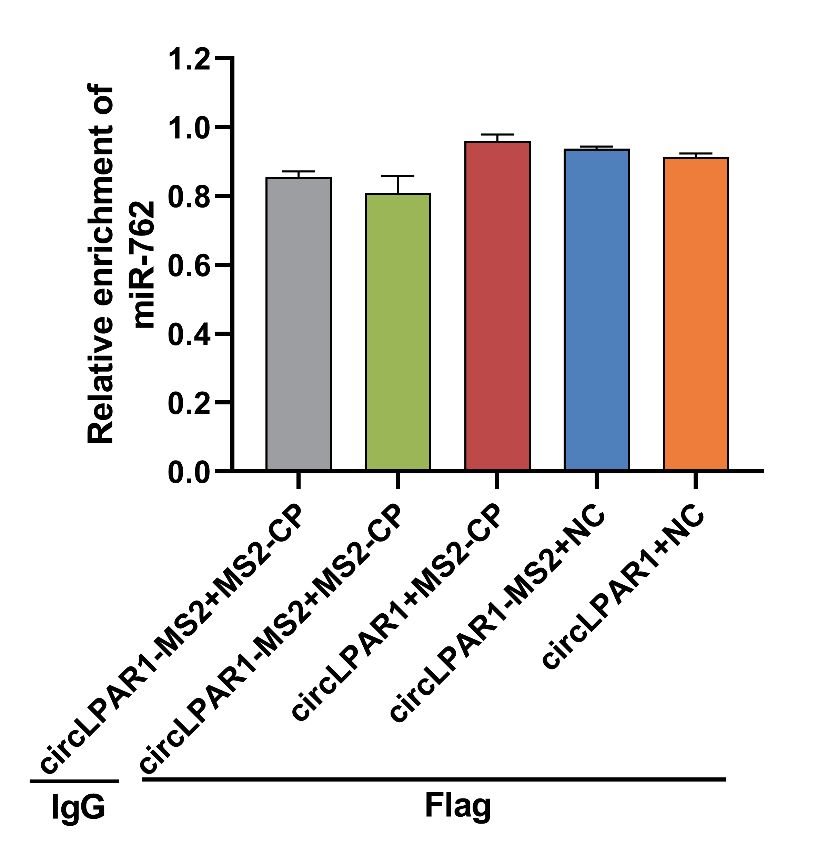
**

Fig. S17. Effect of exosomal circLPAR1 on colorectal cancer progression *in vivo*. (A) The expression of circLPAR1 in tumor tissues from NC, circLPAR1, circLPAR1-Exos and NC-Exos group. Statistical significance was assessed using two-tailed Student’s *t*-test. The values represent the mean ± SD. ^*^*P* < 0.05. (B) Representative images of H&E staining in NC, circLPAR1, circLPAR1-Exos and NC-Exos group. Scale bar, 100 μm.


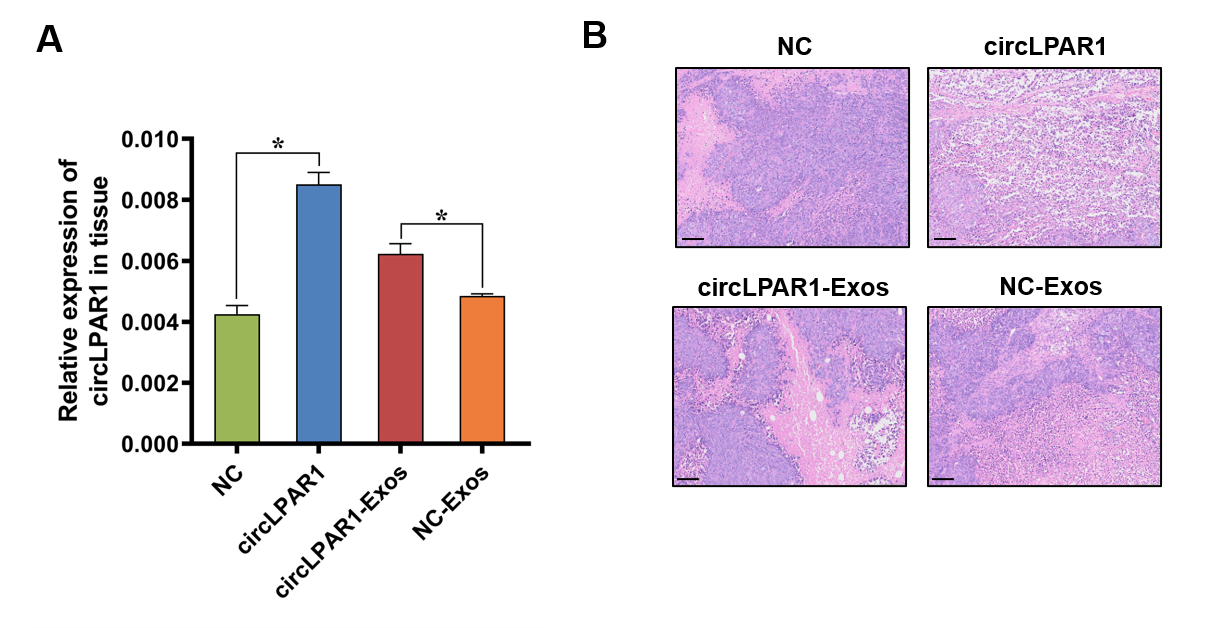

Supplement: Supplementary file 1 — Additional file 1: Supplementary Materials and Methods. Table S1. Demographic and clinical characteristics of the subjects who participated in RNA-Seq and proteogenomic analysis. Table S2. Five differentially expressed circRNAs and significant fold changes. Table S3. Demographic and clinical characteristics of the subjects in FISH analysis. Table S4. Demographic and clinical characteristics of subjects with colorectal cancer and cancer-free controls. Table S5. Characteristics of patients with other types of cancer. Table S6. Demographic and clinical characteristics of the colorectal cancer patients before and after operation. Table S7. Characterization of circLPAR1-binding proteins. Table S8. The correlation between protein and its parental gene expression and prediction of RNA binding ability. Table S9. Sequences of circLPAR1 used for FISH analysis in the study. Table S10. Sequences of primers used for RT-qPCR in the study. Figure S1. The expression of circRNAs detected by RT-qPCR. Figure S2. The expression of circLPAR1 in CRC cells treated with actinomycin D and colorectal tumors. Figure S3. Characterization of exosomes derived from human plasma. Figure S4. The expression of traditional clinical biomarkers in plasma. Figure S5. ROC curve analysis of exosomal circLPAR1 and traditional clinical biomarkers in human plasma. Figure S6. Fluorescence observation of colorectal cancer cells after incubation with PKH67-labelled exosomes or non-exosomes. Figure S7. Fluorescence observation of colorectal cancer cells incubation with PKH67-labelled exosomes pretreated with cytochalasin D. Figure S8. The expression of circLPAR1 in exosomes derived from colorectal cancer cells. Figure S9. The effect of exosomal circLPAR1 on invasion and migration abilities of HCT116 cells. Figure S10. The effect of circLPAR1 overexpression on colorectal cancer cellular phenotypes. Figure S11. The cellular localization of circLPAR1 in colorectal cancer cells. Figure S12. MS2-CP-Flag was pulled do [file 12943_2021_1471_MOESM1_ESM.docx]
